# TREATment of Lower Respiratory Tract Infection in Selected Hospitals in Southern Sri Lanka (TREAT-SL): study protocol for a stepped-wedge, cluster-randomized clinical trial

**DOI:** 10.1186/s13063-026-09628-0

**Published:** 2026-03-18

**Authors:** P. D. W. D. De Zoysa, S. A. Weerasinghe, J. Gamage, M. D. Iglesias-Ussel, S. Olague, A. Obale, J. A. Gallis, D. Palangasinghe, B. Senadheera, S. Vasana, C. Nix, C. K. Bodinayake, A. D. S. Nagahawatte, W. M. D. G. B. Wijayaratne, M. R. P. Kurukulasooriya, M. Premamali, U. H. B. Y. Dilshan, J. Ngocho, T. Østbye, E. B. Laber, M. H. Watt, E. Myers, C. W. Woods, S. Naggie, A. Kenny, H. Chakraborty, L. G. Tillekeratne

**Affiliations:** 1 Department of Medicine, Faculty of Medicine, University of Ruhuna, Galle, Sri Lanka; 2 Duke-Ruhuna Collaborative Research Centre, Faculty of Medicine, University of Ruhuna, Galle, Sri Lanka; 3 Department of Medicine, Duke University School of Medicine, Durham, NC USA; 4 Duke Clinical Research Institute, Duke University, Durham, NC USA; 5 Duke Global Health Institute, Duke University, Durham, NC USA; 6 Department of Microbiology, Faculty of Medicine, University of Ruhuna, Galle, Sri Lanka; 7 Kilimanjaro Christian Medical Centre, Moshi, Tanzania; 8 Department of Family Medicine and Community Health, Duke University, Durham, NC USA; 9 Department of Population Health Sciences, School of Medicine, University of Utah, Salt Lake City, UT USA; 10 Department of Obstetrics and Gynecology, Duke University School of Medicine, Durham, NC USA; 11 Department of Biostatistics and Bioinformatics, Duke University, Durham, NC USA

**Keywords:** LRTI, Protocol, eCDST, Sri Lanka

## Abstract

**Background:**

Lower respiratory tract infection (LRTI) is a leading cause of hospitalization and antibacterial use globally. In low- and middle-income countries (LMICs), limited diagnostic resources contribute to inappropriate antibacterial use for LRTI. The aim of this trial is to determine the impact of an electronic clinical decision support tool (eCDST) on LRTI management in Southern Sri Lanka.

**Methods:**

This is a prospective, open-label, stepped-wedge, cluster-randomized trial that will be conducted at three public hospitals. A total of 765 patients aged ≥ 14 years who are hospitalized with LRTI will be enrolled across 9 clusters; each cluster consists of a pair of wards (1 male and 1 female). The intervention consists of implementing an eCDST (RespiQuestAB) that physicians use on their mobile phones. RespiQuestAB integrates real-time influenza incidence in Sri Lanka with epidemiological and clinical predictors of LRTI etiology to estimate the probability of the following diagnoses: influenza, other viral (including SARS-CoV-2) infection, bacterial infection, noninfectious condition, or indeterminate. Point-of-care (POC) tests for influenza, SARS-CoV-2, *Streptococcus pneumoniae*, or procalcitonin are recommended if a positive result increases the probability of the associated diagnosis by at least 5%; clinicians may also order these tests independently. RespiQuestAB provides treatment recommendations regarding antibacterial and antiviral (oseltamivir) use based on the probability of diagnosis. Participants will be followed longitudinally to assess antibacterial prescription and clinical outcomes.

The co-primary outcomes are as follows: (1) Duration of antibacterial prescription for the index visit and (2) a composite clinical outcome associated with worsening LRTI by day 30. Secondary outcomes include the individual clinical outcomes of the composite outcome, receipt, and duration of antibacterial and antiviral prescriptions and physician adherence to diagnostic tests and treatments recommended by RespiQuestAB. Exploratory outcomes include Desirability of Outcome Ranking (DOOR) level arranged in an ordinal scale, individual DOOR levels, and prescription of immunomodulatory therapy for SARS-CoV-2.

**Discussion:**

Results of this clinical trial will provide information on the impact of the RespiQuestAB on antibacterial use and clinical outcomes among patients with LRTI in an LMIC setting.

**Trial registration:**

ClinicalTrials.gov NCT06331364. Registered on March 25, 2024.

**Supplementary Information:**

The online version contains supplementary material available at 10.1186/s13063-026-09628-0.

## Background

Globally, lower respiratory tract infection (LRTI) is among the leading causes of hospitalization and is a major driver of antibacterial use and overuse [1, 2]. Viral and bacterial LRTI have similar presentations, leading clinicians to overprescribe antibacterials for fear of missing an otherwise lethal bacterial infection or superinfection [3]. Antibacterial overuse is worse in low- and middle-income countries (LMICs), where LRTI remains the leading infectious cause of mortality and where diagnostic capacity is limited [1, 4–6]. However, emerging data from both pediatric and adult cohorts indicate that viral LRTIs are often recognized more commonly than bacterial LRTIs. In many countries, respiratory viruses are commonly observed to cause LRTIs in both children and adults [4, 7–9]. In Sri Lanka, our team has shown that respiratory viruses can be identified in almost 40% of patients hospitalized with LRTI, with the most commonly detected viruses being influenza A, human rhinovirus/enterovirus (HRV/HEV), and respiratory syncytial virus (RSV). In this previous study, 84.4% of children and 87.8% of adult patients with a respiratory virus identified were treated with antibiotics during hospitalization [10]. We have also shown that LRTI is the most common indication for antibiotic use in the inpatient setting in five public hospitals in Southern Province, Sri Lanka [11]. The unnecessary use of antibacterials for treatment of viral infection increases downstream antimicrobial resistance, which is estimated to cause up to 10 million deaths annually by 2050 [12]. Conversely, lack of antibacterial use for bacterial LRTI is associated with increased morbidity and mortality [13]. Thus, optimally targeting antibacterials, with antibacterial prescription for bacterial LRTI and antibacterial avoidance for viral LRTI, is vital in the care of patients with LRTI.

Access to diagnostic test results can reduce inappropriate antibacterial use for respiratory viral infection and help target therapy [14–17]. In some countries, diagnostic tests have been shown to significantly reduce antibacterial prescriptions for viral respiratory infections [17, 18]. In Sri Lanka, our team has shown that a positive rapid influenza test was associated with a 20% reduction in antibacterial prescriptions (84% versus 62%) among patients with influenza [18]. However, limited availability of diagnostics continues to drive inappropriate antimicrobial use for LRTIs [3, 19]. Pathogen-based tests, like sputum or blood cultures and multiplex polymerase chain reaction (PCR) of nasopharyngeal samples, may have limited sensitivity, cover few organisms, and fail to distinguish colonization from infection [19]. Additionally, detection of a pathogen from the upper respiratory tract may not indicate the etiology of infection in the lower respiratory tract [3]. In LMICs, the use of existing diagnostics is further hampered by lack of access and high cost [20, 21]. Given limitations associated with pathogen-based tests, host-based diagnostics, which broadly classify infections as bacterial or viral, may provide valuable adjunctive information in diagnosing and managing antimicrobials for respiratory infection [22, 23]. Host biomarkers act as surrogate measures of the immune response to infection and do not depend on identifying a specific pathogen [24, 25]. C-reactive protein (CRP) and procalcitonin (PCT) are the most widely studied biomarkers to date for classifying viral versus bacterial respiratory infection [26, 27].

Evidence-based algorithms for the diagnosis and treatment of LRTI are not widely available in high-income or LMIC settings. Especially in LMICs, where access to diagnostics may be limited, strategies based on local epidemiology are critical for maximizing impact and minimizing cost. For LMICs, the Integrated Management of Childhood Illness (IMCI) and Integrated Management of Adolescent and Adult Illness (IMAI) developed by the World Health Organization (WHO) provide basic strategies on diagnosing and treating conditions such as LRTI [28–30]. These algorithms rely on syndromic definitions that can be applied broadly in resource-limited settings and have been associated with better health worker performance and quality of care [30]. However, the IMCI/IMAI do not capture local epidemiology of infection [31]. In addition, these guidelines were designed to maximize sensitivity for bacterial infection over specificity, with the result that more patients than necessary may receive antibacterials [32]. New strategies for diagnosing acute febrile illness (AFI) in resource-limited settings are starting to be explored. Pokharel et al*.* demonstrated that serial point-of-care (POC) testing for AFI causes may be more accurate than simultaneous testing in India and Cambodia [33]. A meta-analysis of three randomized clinical trials conducted in Burkina Faso, Ghana, and Uganda among patients with acute febrile illness showed that a diagnostic algorithm combined with POC tests (Malaria Rapid Diagnostic Test (RDT), CRP, white blood cell (WBC) total count and differential counts) could possibly reduce inappropriate antibacterial prescription without impairing patient outcomes [34]. In Afghanistan and Nigeria, ALMANACH (Algorithm for the Management of Acute Childhood Illness), a digital IMCI tool, reduced antibiotic prescriptions for children [35]. Keitel et al*.* developed a novel electronic algorithm (e-POCT) including POC tests (pulse oximetry, glucometer, hemoglobin, malaria test, rapid HIV, CRP, and PCT) to triage children < 5 years with AFI; e-POCT resulted in non-inferior clinical outcomes and decreased antibacterial use compared to ALMANACH [36]. These strategies all emphasize the need for evidence-based algorithms, as etiology of infection as well as performance of biomarkers may vary based on local context [22].

### Rationale

Duke University and Ruhuna University have had a formal research collaboration (Duke–Ruhuna Collaborative Research Center) since 2006. Multiple large cohort studies in infectious disease epidemiology have been conducted in the Southern Province, Sri Lanka, through this collaboration. Over the past decade, our study team has demonstrated that LRTI is the most common reason for antibacterial use in the inpatient setting in Southern Sri Lanka [11]. In addition, we have shown that viruses such as influenza, RSV, and adenovirus are more commonly identified than bacteria as a cause of LRTI in Southern Sri Lanka [10].

In this study, we will leverage the rich body of epidemiologic information we have gathered in past years to develop and implement a tool to improve the diagnosis and management of LRTI, using Southern Sri Lanka as our initial test region. By conducting this interventional trial in the same setting as that in which we generated the initial data, we hope to produce a tool that is evidence based, locally relevant, culturally appropriate, and acceptable to local physicians.

The TREATment of Lower Respiratory Tract Infection in Sri Lanka (TREAT-SL) trial will evaluate the impact of a novel electronic clinical decision support tool (eCDST), known as RespiQuestAB, for managing LRTI. The rationale for the trial is that an evidence-based tool consisting of real-time surveillance data, clinical predictors, POC pathogen tests, and POC biomarker tests will result in improved management of LRTI.

Our hypothesis is that the use of the eCDST will reduce antibacterial prescription and result in non-inferior clinical outcomes.

## Materials and methods

### Objectives

The primary objective of this study is to determine the impact of RespiQuestAB on clinical outcomes and antibacterial prescription in subjects with LRTI in the intervention group compared to the control group. The secondary objectives include the following: To compare clinical outcomes at discharge and by day 30 in the intervention and control groups; to compare the use of and duration of use of advanced care and therapies in the intervention and control groups; to compare the duration of hospitalization in the intervention and control groups; to compare antibacterial, oseltamivir, and SARS-CoV-2 antiviral prescription and duration of prescription between the intervention and control groups; and to determine physicians’ adherence to RespiQuestAB diagnostic test and treatment recommendations. The exploratory aims are to compare ordinal clinical outcomes by day 30 in the intervention and control groups when using a Desirability of Outcome Ranking (DOOR) scale and to compare SARS-CoV-2 immunomodulator prescription and duration of prescription between the intervention and control groups.

### Study setting

TREAT-SL will be conducted in three public hospitals in the Southern Province of Sri Lanka: National Hospital Galle (NHG), District General Hospital Matara (DGM), and Base Hospital Balapitiya (BHB). NHG is an 1800-bed hospital in Galle district and the largest tertiary care center in the southern province. The hospital is affiliated with the Faculty of Medicine, University of Ruhuna. DGM is the largest hospital in Matara district, Sri Lanka, with approximately 1000 beds. BHB is the largest base (secondary) hospital in Galle district, Sri Lanka, and has approximately 500 beds.

### Trial design

**Fig. 1:**
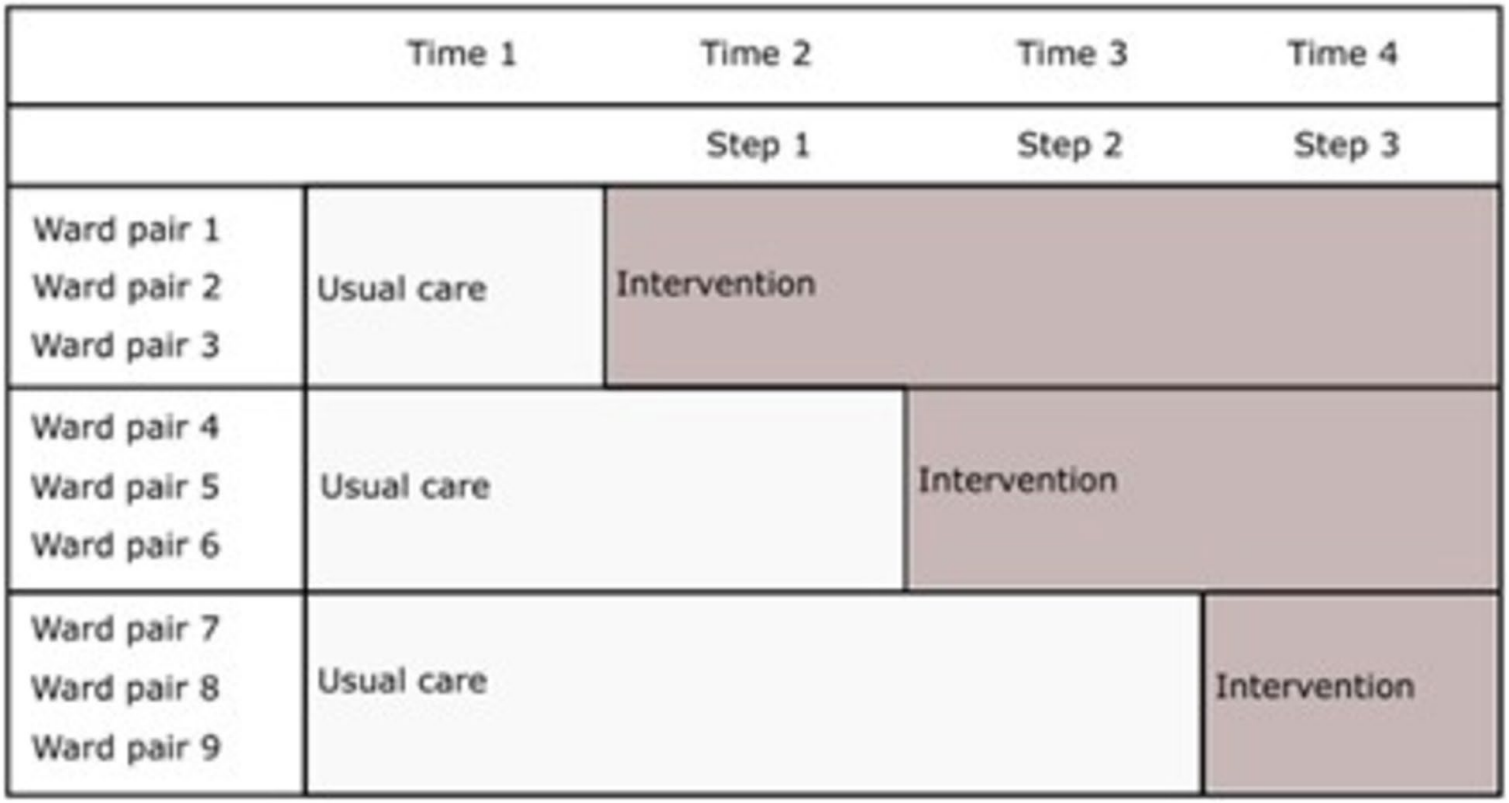
Stepped-wedge, cluster-randomized study design displaying the clusters (ward pairs) and randomization to intervention

This study is an open-label, stepped-wedge, cluster-randomized trial. The stepped-wedge design was chosen to increase logistical feasibility, increase participation in the study (since all clusters will eventually receive the intervention), reduce contamination bias, and reduce the risk of a cluster dropping out of the study because it is not receiving the intervention. This trial includes nine clusters (five in NHG, two in DGM, and two in BHB), with three clusters randomized to intervention at each step/time period until all clusters receive the intervention. All clusters (pairs of wards) will be observed for four time periods, each of which will last between 3 and 6 months, with the time period ending when the target sample size is reached for that period. Participant sampling will be done on a repeated cross-sectional basis. The study design is depicted in Fig. 1.

### Study population

The study will enroll children ≥ 14 years of age and adults admitted with LRTI to the medical wards at three public hospitals in Southern Province, Sri Lanka.

#### Participant inclusion criteria

In order to be eligible to participate in this study, an individual must meet all of the following criteria:

1. Admitted within prior 48 h
2. Have evidence of new acute respiratory illness (< 14 days of symptoms), as indicated by at least one of the following:
  - New cough or sputum production
  - Chest pain
  - Dyspnea or tachypnea (respiratory rate > 20 breaths/min)
  - Abnormal lung examination
3. Have evidence of acute infection, as indicated by at least one of the following:
  - Self-reported fever or chills
  - Documented fever ≥ 38 °C (100.4 °F)
  - Documented hypothermia < 35.5 °C (95.9 °F)
  - Leukocytosis (white blood cell count > 10,000/µL)
  - Leukopenia (white blood cell count < 3000/µL)
  - New altered mental status
4. Ability and willingness of patient, parent, or legally authorized representative (LAR) to give informed consent
5. Ability of children 14–17 years of age to provide assent
6. Ability to complete follow-up encounter at 30 days in person or by telephone

#### Participant exclusion criteria

Participants who meet any of the following criteria will be excluded from enrollment in this study:

1. Hospitalized recently (within last 28 days)
2. Enrolled into this clinical trial previously
3. Surgery in the past 7 days
4. Unable or unwilling to complete the follow-up encounter
5. Likely to be transferred from the medical wards within 24 h of enrollment (to another ward or to another hospital)
6. Has underlying conditions or circumstances for which physicians would be unlikely to withhold antibacterials:
  - Vasopressor therapy
  - Cystic fibrosis
  - Known severe immunosuppression
    i. Cancer or another condition with neutropenia (absolute neutrophil count < 1000/µL)
    ii. Solid-organ or hematopoietic stem-cell transplant within the previous 90 days
    iii. Active graft-versus-host disease or bronchiolitis obliterans
    iv. On chronic steroids equivalent to prednisone 20 mg daily for ≥ 2 weeks or other targeted cytotoxic or biologic immunosuppressants within the prior 4 weeks
    v. Human immunodeficiency virus infection with a CD4 cell count < 200/µL)
  - Has an accompanying non-respiratory infection
  - Has evidence of a lung abscess or empyema [7, 37]
  - Has respiratory failure at enrollment, evidenced by use of non-invasive or invasive ventilation

### Participant recruitment and retention

Participants meeting the LRTI case definition will be approached by trained research staff within 48 h of hospital admission to medical wards (as opposed to the emergency treatment units or outpatient departments), since clinical decisions during hospitalization are made by ward physicians. The study will be explained in English or the local languages of Sinhala or Tamil by multilingual, trained research assistants.

Potential participants will be screened for eligibility by study personnel during hospital admission. If the patient meets all inclusion criteria and does not meet any exclusion criteria, they will be offered enrollment following informed consent. Written, informed consent in Sinhala, Tamil, or English will be obtained from all adult patients and the parents or guardians of patients 14–17 years of age. Assent will be obtained from patients 14–17 years of age.

Retention will be enhanced by several methods. Participants will be provided with the option of following up by either telephone or in person, depending on preference and their available resources. There will be a 3-day grace period for the 30-day follow-up visit, during which participants can follow up.

Treating physicians who are practicing in the wards during the usual care or intervention (RespiQuestAB) phases will not be considered investigators or study participants. They will only use RespiQuestAB to obtain additional diagnostic and treatment information recommendations when treating patients with LRTI in wards that are in the intervention phase.

### Randomization

Medical wards within each hospital will be randomized in pairs (clusters) to the intervention. All nine pairs of wards (clusters) will start with an initial period of usual care. At intervals of 3–6 months (steps), a set of three clusters will be randomized to use the intervention until all pairs of wards have crossed over, as shown in Fig. 1.

The pairs of wards comprising a cluster will be predefined, according to existing hospital structure in which one male and one female ward are paired and share medical staff. If the minimum number of participants needed to be enrolled is not met for a certain cluster within the 3-month time interval, enrollment will be extended for a maximum of 6 months per interval for all clusters. If the minimum number of participants needed to be enrolled is met earlier than 3 months for a certain cluster, enrollment in that cluster will continue until twice the target number for the cluster is enrolled or until the next time interval is met, whichever comes sooner. If twice the target number for the cluster is enrolled prior to the next time interval, enrollment into that cluster will be paused until the next time interval.

The sequence of allocation of the pairs of wards to the sequential implementation of the algorithm will be prepared by an unblinded statistician using a computer-generated random number table using R software (version 4.4.2). A constrained randomization scheme will be used to ensure that for each of the two smaller hospitals, the two clusters from that hospital do not end up in the same sequence (in order to maximize balance of possible hospital-level effects). This unblinded statistician will communicate the allocation sequence to the study team. Training for implementing the intervention will be conducted in the appropriate wards no sooner than 2 weeks before the intervention is implemented. Wards will be blinded to their randomization time point until training for the implementation of RespiQuestAB begins on the ward, which will not be more than 2 weeks prior to the switch. During these 2 weeks, an orientation session regarding the study objectives, protocol, RespiQuestAB use, and ethical considerations will be provided to clinicians. Due to the nature of the intervention, the treating clinicians, study staff, and participants will not be blinded to allocation.

### Study procedures

The study procedures common to both groups at the initial visit will include administering a questionnaire and reviewing the medical record to obtain baseline information about sociodemographics, chronic medical conditions, medication use, vital signs, physical exam findings, and collection of samples, including 15 mL of blood, one nasal or nasopharyngeal sample, sputum sample (for future research), and a urine sample. All participants will be followed throughout their hospitalization, and information about test results, antimicrobials prescribed and received, and clinical outcomes will be recorded. All participants will participate in a follow-up visit (by telephone or in person) at day 30 ± 3 days to obtain information about antimicrobial use, clinical outcomes, and post-discharge resource utilization. Home visits may be conducted at the participants’ homes to complete the follow-up encounter, if needed. No specific posttrial care provisions will be provided, as participants will remain in the study only until the day-30 follow-up. Free care is available at the three public hospitals, as it is to all patients.

**Fig. 2:**
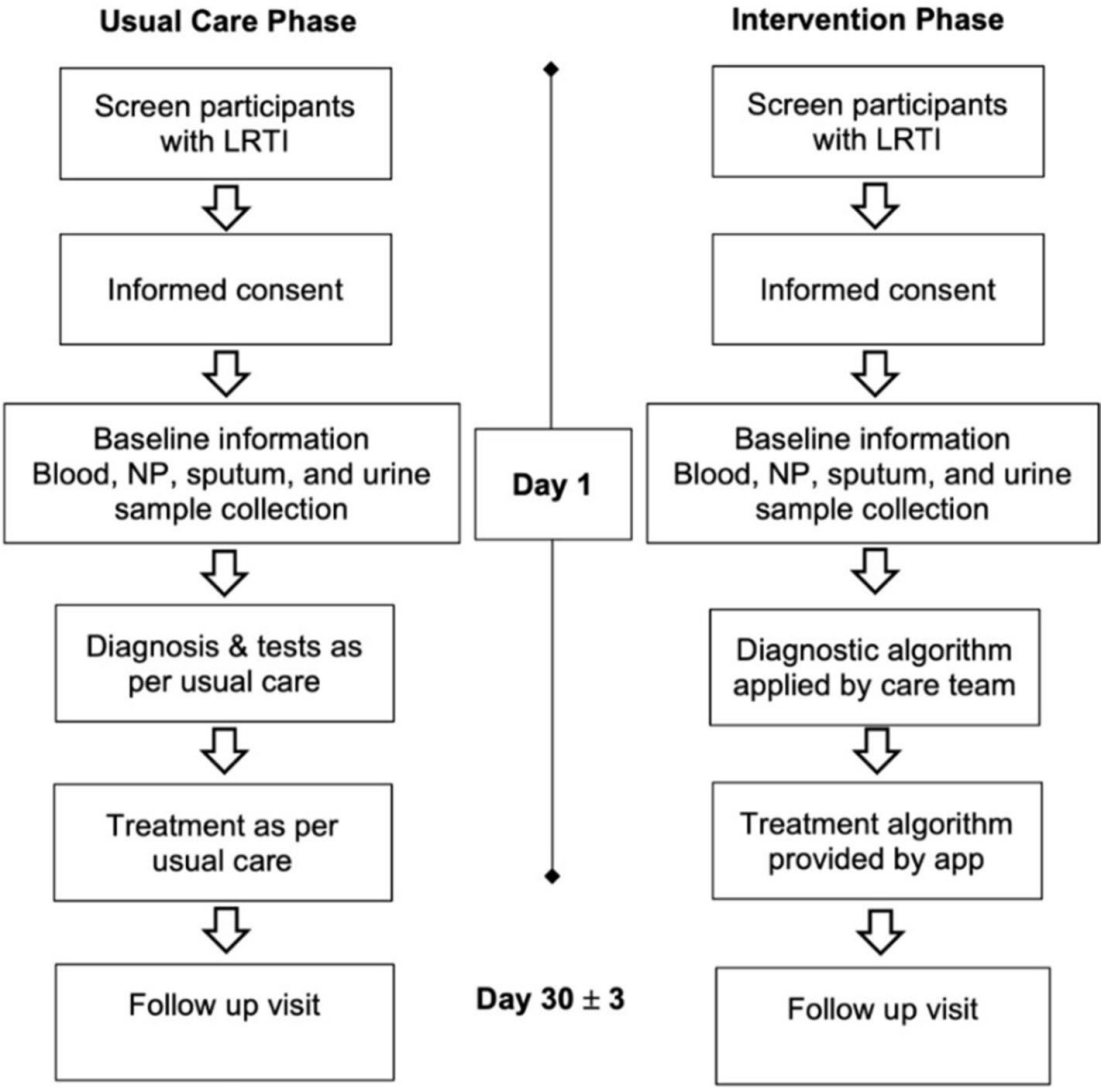
Schematic of study procedures followed by patients during the usual care and intervention phases in the TREAT-SL trial

Treat-SL consists of two treatment conditions: usual care and intervention (Fig. 2).

#### Usual care

Study staff will inform the treating clinicians to diagnose and treat patients according to usual practice. Clinicians will be able to order routine diagnostic testing as per standard practice. These tests may include complete blood count, chemistries, CRP testing, erythrocyte sedimentation rate (ESR) testing, blood and sputum cultures, and chest x-ray or chest CT imaging. Apart from the intervention, participants in both arms will receive usual care as per the treating clinician. Therefore, participants will be allowed to receive any treatment or therapies prescribed by the clinician.

#### Intervention

**Fig. 3:**
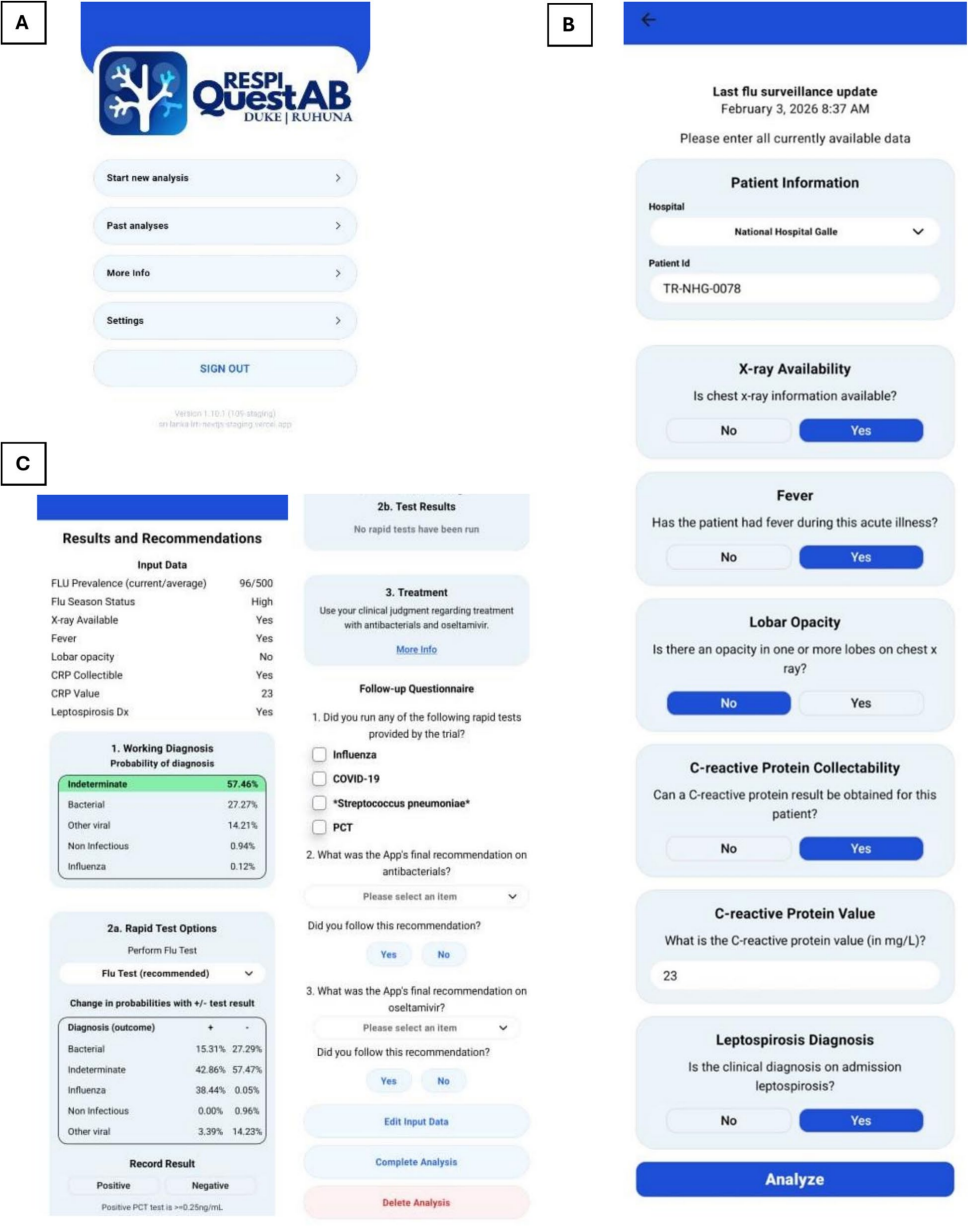
**A** Initial screen of the electronic application, RespiQuestAB **B** home/main screen, and **C** results and recommendations screen

The intervention will involve the use of the eCDST, known as RespiQuestAB, for diagnosing and managing LRTI. RespiQuestAB is an electronic application (depicted in Fig. 3) that physicians will be asked to download onto their smartphones.

RespiQuestAB implements a pretrained machine learning algorithm and takes into account real-time incidence of influenza in Sri Lanka (updated weekly by study team), as well as epidemiological and clinical variables that are predictive of LRTI etiology, based on a biorepository of LRTI data we previously created in Sri Lanka. A maximum of 25 epidemiological/clinical variables are included in the algorithm, including variables such as the presence of fever, number of days of cough, and white blood cell count. On average, clinicians will be asked to respond to approximately 10 of these questions about a patient’s presenting signs and symptoms. Based on the responses, RespiQuestAB may advise performing POC tests for influenza, SARS-CoV-2, *Streptococcus pneumoniae*, and/or PCT if a positive test result would increase the probability of the most likely diagnosis by at least 5%. Clinicians will also have the ability to perform these POC tests of their own accord and will be able to see post-test probabilities based on the test result prior to performing the test. Given the need for additional POC testing as per the sequential RespiQuestAB diagnostic section instructions, there may be an additional collection of a blood specimen (5 mL maximum) and/or up to two additional nasal or nasopharyngeal samples within 48 h of enrollment. Based on inputs into RespiQuestAB, the tool will provide a probability of influenza, other viral infection (including COVID-19), bacterial infection, noninfectious condition, or indeterminate condition. Clinicians will then be provided with a treatment recommendation (treatment with antibacterials or oseltamivir or withholding of these drugs) based on the most likely diagnosis. POC testing as advised by RespiQuestAB will be conducted by either clinical staff or trained research staff. If performed by research staff, results will be delivered on paper or electronically/by telephone within 6 h of testing to ≥ 2 primary clinicians on the team (including the consultant/attending-level physician or senior registrar). Since NHG, DGM, and BHB do not have an electronic medical record system, clinicians will be asked to input the results from diagnostic testing and to follow the treatment recommendations as per RespiQuestAB but will be advised that the final decision regarding prescription of antimicrobials is entirely at their discretion.

The principal investigators (PIs), co-investigators, and research team members will be responsible for overseeing compliance with all study parameters from enrollment to discharge. After discharge, a designated research assistant will be in touch with the participant or guardian to ensure completion of the follow-up assessment on day 30.

Adherence to use of RespiQuestAB will be monitored through information collected on the case report form by the research assistants. The research assistants will document all details required to assess adherence, such as diagnostic tests recommended by the eCDST, whether the recommended tests were performed, the test results, and whether the treatment recommendations were followed. In addition, the treating physicians will respond to questions in the RespiQuestAB app after each case to indicate whether they conducted the recommended diagnostic tests and whether they followed the treatment recommendations.

Physician adherence to RespiQuestAB will be enhanced through strategies such as providing initial training via a workshop, minimizing data entry into the app (approximately 10 variables), providing compensation for Internet access, and addressing barriers in real time by the site PI.

During the intervention, use of the intervention may be discontinued for a given trial participant by the PI due to any clinical adverse event, laboratory abnormality, or other medical condition or situation if continued participation in the study would be detrimental to the participant. Participation may also be discontinued if the participant meets an exclusion criterion (either newly developed or not previously recognized) that precludes further study participation. Moreover, the study may be discontinued for a given participant upon participant/guardian request or by withdrawal of the informed consent.

**Fig. 4:**
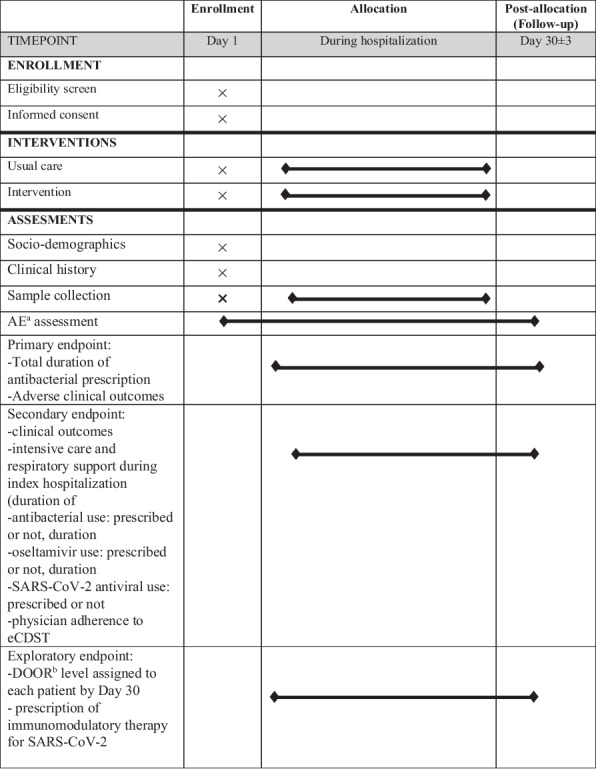
Schedule of enrollment, interventions, and assessments

Figure 4 presents a detailed participant timeline, developed in accordance with SPIRIT guidelines [38].

### Outcome measures

The primary outcome will consist of a co-primary endpoint: (1) Total duration of antibacterial prescription for the index visit (superiority analysis) and (2) clinical outcomes by day 30 (non-inferiority analysis). Total duration of antibacterial prescription for the index visit will include the number of days that antibacterials are prescribed during the index hospitalization, as well as the number of days that antibacterials are prescribed at discharge from the hospital (intended use), for a maximum total of 14 days. The time of assessment will be 30 days (after enrollment) or at discharge. A day of antibacterial prescription will be defined as each day a subject is prescribed any oral, intramuscular, or intravenous antibacterial (may be nonconsecutive days), excluding antivirals, antifungals, or anti-parasitics.

The primary, secondary, and exploratory outcomes of the TREAT-SL trial are listed in Table 1.

**Table 1:** Primary, secondary, and exploratory outcomes of the TREAT-SL trial

**Category**	**Outcome**	**Details**
**Primary outcome (co-primary)**	Total duration of antibacterial prescription for the index visit	Superiority analysis, includes number of days that antibacterials are prescribed during index hospitalization and at discharge (maximum 14 days), and excludes antivirals, antifungals, anti-parasitics
	Clinical outcomes by day 30	Non-inferiority analysis and composite binary endpoint (any of the following): (a) use of noninvasive ventilation (CPAP^a^ or BiPAP^b^), (b) use of mechanical ventilation or ECMO^c^, (c) readmission to hospital, and (d) death
**Secondary outcomes**	Individual components of the composite clinical endpoint	Each of the four adverse outcomes listed in primary outcome 2
	Seeking outpatient care and receiving antibacterial prescription by day 30	At day 30 only
	ICU^d^ admission and duration during index hospitalization	Admission to ICU and duration in days
	Use and duration of use of supplemental oxygen during index hospitalization	Via nasal cannula, high-flow nasal cannula, or face mask
	Duration of noninvasive ventilation	During index hospitalization
	Duration of mechanical ventilation	During index hospitalization
	Antibacterial prescription	On days 1, 2, and 3, at discharge, and by day 30
	Total antibacterial exposure	Total number of days antibacterials were prescribed during hospitalization and by day 30
	Oseltamivir prescription	On day 1, day 2, day 3, at discharge, and by day 30
	Total oseltamivir exposure	Total number of days oseltamivir is prescribed during hospitalization and by day 30
	SARS-CoV-2 antiviral prescription timing	Day 1, day 2, day 3, at discharge, and by day 30
	Physician adherence to eCDST^e^ diagnostic recommendations	Within 24 h and 48 h (intervention group only)
	Physician adherence to eCDST treatment recommendations	Within 24 h and 48 h (intervention group only)
**Exploratory outcomes**	DOOR (desirability of outcome ranking) level by day 30	Ordinal scale (see Table 2)
	Individual DOOR levels	By day 30
	Prescription of immunomodulatory therapy for SARS-CoV-2	Dexamethasone or tocilizumab on day 1, day 2, day 3, and at discharge

^a^Continuous positive airway pressure

^b^Bilevel positive airway pressure

^c^Extracorporeal membrane oxygenation

^d^Intensive care unit

^e^electronic clinical decision support tool

The clinical endpoint will be a binary endpoint that is a composite of adverse outcomes that could be attributed to withholding antibacterials. These outcomes would not be present at enrollment and could occur anytime until day 30. At least one of the following adverse outcomes should be present to meet this endpoint: (1) Use of noninvasive ventilation (i.e., continuous positive airway pressure [CPAP] or bilevel positive airway pressure [BiPAP]) for treatment of the acute illness, (2) use of mechanical ventilation (via endotracheal tube) or extracorporeal membrane oxygenation (ECMO), (3) readmission to hospital, or (4) death.

Day 1 will be the day of enrollment, and participants will be followed through day 30.

Secondary outcomes will include the following: Individual components of the binary clinical outcomes in the co-primary endpoint, receipt and duration of antibacterial and antiviral prescriptions, and physician adherence to the RespiQuestAB diagnostic and treatment recommendations in the intervention group.

The exploratory outcomes will be the following: the Desirability of Outcome Ranking (DOOR) level by day 30 arranged in an ordinal scale as shown in Table 2, individual DOOR levels by day 30, and prescription of immunomodulatory therapy for SARS-CoV-2 (i.e., dexamethasone, tociluzumab) on day 1, day 2, day 3, and at discharge. Qualifying symptoms for adequate clinical improvement per the DOOR are shown in Table 3, and solicited adverse events per the DOOR are shown in Table 4.

**Table 2:** Desirability of Outcome Ranking (DOOR) of clinical outcomes (from most to least desirable)

	Adequate clinical improvement (assessed at day 30)*	Solicited adverse events (assessed through day 30)+
1	Yes	None
2	Yes	Mild-severe (grades 1–3)
3 4	No No	None Mild-severe (grades 1–3)
5	No, with outpatient visit for LRTI	None or any grade
6	No, with subsequent hospitalization	None or any grade
7	Death (any cause)	–

*Adequate clinical improvement includes *all* of the following: Improvement in at least one qualifying symptom present at enrollment (see Table 3) by at least one step. The absence of deterioration in any qualifying symptom (see Table 3), defined as worsening by at least one step of any qualifying symptom

No medically attended visit for LRTI

+Solicited adverse events include antibiotic-related side effects, as listed in Table 4

**Table 3:** Qualifying symptoms for adequate clinical improvement and grading scale

	Mild	Moderate	Severe
Cough	Occasional coughing (less than hourly)	Frequent coughing (one or more times an hour and interfered with activity or sleep)	Almost constant coughing (never free of cough or need to cough, made activity or sleep nearly impossible)
Sputum production	Noticeable as a problem but did not interfere with activity	Caused a great deal of inconvenience	An almost constant problem
Chest pain	Noticeable only when coughing	Noticeable during deep breaths and when coughing	Almost constant, present even when resting, without cough
Difficulty breathing	Noticeable during strenuous activity, such as going up a flight of stairs or walking more than a block on level ground	Noticeable during light activity or when washing or dressing	Almost constant, present even when resting

**Table 4:** Solicited adverse events and grading scale

	Mild	Moderate	Severe
Abdominal pain	Mild or intermittent and did not interfere with daily activity	Moderate or persistent and interfered with daily activity but did not necessitate a medical visit	Prevented daily activity and resulted in medical visit
Vomiting	1 episode/day	2–3 episodes/day but did not necessitate a medical visit	Prevent daily activity and resulted in medical visit/hospitalization
Diarrhea	Looser than normal stools occurring 3–6 times/day	Looser than normal stools > 6 times/day but did not necessitate a medical visit	Bloody diarrhea or diarrhea that required clinical evaluation or hospitalization
Allergic reaction	New localized rash or itching without rash	New diffuse rash covering multiple areas of the body but did not necessitate a medical visit	New rash requiring clinical visit
Candidiasis	Mild mucocutaneous candidiasis, with no treatment	Moderate mucocutaneous candidiasis and interfered with daily activity but did not necessitate a medical visit	Severe mucocutaneous candidiasis resulted in medical visit

### Sample size

The trial is powered on both co-primary endpoints. For co-primary endpoint 1 (total duration of antibacterial prescription for the index visit), power calculations were performed using the R package swCRT design [39]. We assume that nine pairs of wards (clusters) will cross over to intervention in three steps (3 pairs of wards per step), leading to four total time periods (including baseline). We assume a type 1 error of 0.025 since this is a co-primary endpoint. We assume a standardized effect size ($\delta$) of 0.5. Based on prior data, we estimate the mean antibiotic days to be 3.6 (SD = 3.9). Given this standard deviation, we estimate that the unstandardized effect size ($\Delta$) would be $\frac{\Delta }{SD}=\delta \Rightarrow\Delta =\delta \times SD\Rightarrow\Delta =3.9\times 0.5=$ 1.95 antibiotic days. Under these assumptions, we would have 81% power to detect this effect with 765 total subjects recruited, assuming 20% of subjects drop out of the study.

For co-primary endpoint 2 (composite of adverse outcomes that could be attributed to withholding antibacterials), power calculations were performed using the non-inferiority calculator in the artbin package in Stata. Since there are no non-inferiority power calculators for stepped-wedge trials, we calculate non-inferiority under an individually randomized trial design and inflate the sample size for a design effect defined by an ICC of 0.05 and nine clusters. We assume a type 1 error of 0.025 since this is a one-sided test. Under these assumptions, we have 90% power to conclude non-inferiority with a control period rate of 10% and a 10% non-inferiority margin with 765 total participants, assuming a 20% dropout.

### Data management

Participant confidentiality is strictly held in trust by the participating investigators, their staff, and the sponsor(s) and their agents. The study participant’s contact information will be securely stored at each clinical site for internal use during the study. At the end of the study, all records will continue to be kept in a secure location for at least 5 years beyond the conclusion of the study.

Study participant research data, which is for purposes of statistical analysis and scientific reporting, will be entered onsite into a secure Research Electronic Data Capture (REDCap) database and stored in a HIPAA-compliant online drive or secure cloud storage. These data will not include the participant’s contact or other identifying information. Rather, individual participants and their research data will be identified by a unique study identification number. The study data entry and study management systems used by clinical sites and by the coordinating center will be secured and password protected. At the end of the study, all study databases will be de-identified and archived in a secure cloud storage under the supervision of the PI.

Samples and data will be stored using codes assigned by the investigators. Data will be kept in password-protected computers. Only investigators will have access to the samples and data. With the participant’s approval and the local IRB approval, samples will be stored at the University of Ruhuna and may be used for future research on the etiology of infection and response to infection.

### Statistical analysis

The primary analysis population will be the intention-to-treat population (based on whether a patient is enrolled from a cluster that is in usual care or intervention phase). A secondary per-protocol analysis will also be performed, based on whether patients receive usual care or intervention-based care.

The co-primary endpoint of total duration of antibacterial prescription for the index presentation will be treated as continuous and will be analyzed using a mixed-effects model, to take into account dependence by time and by pairs of wards (clusters). Based on the expected impact of RespiQuestAB on early antibacterial therapy and based on statistical considerations, we chose to use a maximum of 14 days for total duration of antibacterial therapy. A truncated window was chosen for logistical practicality, clinical significance, and to restrict the distribution of the outcome such that outliers do not overinflate the variance leading to reduced power. The statistical model will account for possible changes in time trend, include a fixed effect for the intervention, and include random intercepts for both the cluster (pairs of wards) and cluster-by-time interaction. The coefficient and confidence interval on the fixed effect for intervention will be used to assess effectiveness. The statistical model will also include random intercepts for both the cluster (pairs of wards) and cluster-by-time interaction. Both co-primary endpoints will be assessed at type 1 error of 0.025 to take into account multiple testing. Additionally, prior data indicates that we can expect about 18% of the patients to have zero antibiotic days. As a sensitivity analysis, we will also analyze this co-primary endpoint using a zero-inflated negative binomial mixed effects model with the same specification described above.

The co-primary endpoint of clinical outcomes (composite of adverse outcomes that could be attributed to withholding antibacterials) by day 30 will be treated as binary and will be analyzed using a regression model that similarly accounts for the dependent data structure. The statistical model will similarly account for possible changes in the underlying time trend and will include a fixed effect for the intervention, with the confidence interval on the fixed effect for intervention being used to assess non-inferiority. The intervention will be considered non-inferior to the control if the upper 97.5% confidence interval is less than the non-inferiority margin of 10%. Both co-primary endpoints will be assessed at type 1 error of 0.025 to take into account multiple testing.

All secondary and exploratory endpoints that are continuous will be analyzed using the same type of statistical model as is used to analyze the co-primary endpoint of total duration of antibacterial prescription. All secondary and exploratory endpoints that are binary will be analyzed using the same type of statistical model as is used to analyze the co-primary endpoint of clinical outcomes by day 30. Ordinal outcomes will be analyzed using an ordinal regression approach in the GEE framework. Secondary and exploratory endpoints will be assessed using only coefficients and confidence intervals.

Although this study is a stepped-wedge cluster randomized trial (i.e., a different set of patients is followed up at each time point within each cluster), we anticipate some missing data on the primary endpoints since the patients will be followed for 30 days. The mixed-effects model is valid under the missing at random framework. For both the mixed effects and GEE models, we will take into account missing data by first examining if there are any baseline (i.e., participant characteristic) variables associated with loss to follow-up. Then, we will adjust for these variables in the statistical models. As an additional sensitivity analysis, we will also adjust for any participant-level or cluster-level characteristics that are imbalanced by time that the clusters cross over.

Subgroup analyses will be conducted to examine the differential effect of intervention on the co-primary endpoint of total duration of antibacterial prescription by sex, baseline disease severity, age group, duration of symptoms before randomization, and the presence of comorbidities. Effect modification will be assessed by adding main effects and interactions between the aforementioned baseline covariates and intervention. A forest plot will be created to visually display the intervention effects within each level of the subgroup.

### Oversight and monitoring

The coordinating center (CC) will be at the Duke Clinical Research Institute (DCRI). The CC functions as a clinical trial center and is responsible for project management and oversight of all committees and working groups in the USA, development of the protocol and all amendments, quality control, monitoring of study progress, and leadership in data analysis, presentations, and publications.

Trial oversight will be conducted by members of the core research team. This steering committee is comprised of investigators from both the USA and Sri Lanka, including physicians, the blinded statistical team, and research coordinators/staff. This steering committee will meet biweekly via Zoom to review trial progress, recruitment status, protocol adherence, and data quality issues. Prior to the steering committee meetings, data for key variables related to study monitoring and progress will be collated using an R script that runs on data inputted into the REDCap database. These data will be presented and discussed during the steering committee meetings.

Day-to-day trial operations are managed by a local project coordinator who is responsible for regulatory compliance, coordination and supervision of research assistants, monitoring participant recruitment, and providing regular updates to the principal investigators and other team members.

The site PI will oversee staff training, ensure adherence to the study protocol, and oversee monitoring of study activities.

### Safety considerations

We will capture the major potential adverse events related to antibacterial withholding in the safety component of the co-primary endpoint. The adverse outcomes that comprise the primary safety endpoint will be included in endpoint reporting, and thus will not be reported a second time as adverse events (AEs). However, those that are categorized as serious adverse events (SAEs, see below) will be reported. Furthermore, the following additional events (not listed under the co-primary endpoint) will be captured and reported as AEs: the development of lung abscess/empyema and the development of pneumonia in non-pneumonia LRTI.

An AE will be defined as any untoward medical occurrence in a participant temporally associated with the study intervention or other aspects of study participation, but not necessarily considered related to the participant’s participation in the research study. An SAE will be defined as an untoward medical occurrence that (1) results in death or (2) is life-threatening. Life-threatening will be defined as the following events: The development of septic shock (requiring vasopressor use), the need for mechanical ventilation, or the need for ECMO.

All adverse events (AEs and SAEs), as described above, occurring after study enrollment until the study day 30 follow-up visit will be recorded.

To oversee the TREAT-SL clinical trial, a Data Safety Monitoring Board (DSMB) will be established as an independent committee responsible for ensuring the safety of research subjects. It is anticipated that the DSMB will meet every 6 months to review the accumulating data. The DSMB would be asked to offer proper perspective on any therapeutic or diagnostic testing advances that may occur during the course of the trial. The DSMB will be comprised of a physician from Sri Lanka, a physician from the USA, and a statistician. The DSMB will be independent from the sponsor and competing interests. A charter has been formed and appended as an annexure (Additional file 1).

### Quality assurance and quality control

Quality assurance personnel may conduct audits at the study sites. Audits will include, but not be limited to, examination of the audit trail of data handling and processes, standard operating procedures, the presence of required documents, and the informed consent process. The investigators agree to accommodate and participate in audits conducted at a reasonable time and in a reasonable manner.

Regulatory authorities from Sri Lanka or elsewhere may also inspect the study site during or after the study. The study site should contact the study leadership immediately if this occurs and must fully cooperate with governmental audits conducted at a reasonable time and in a reasonable manner.

The study leadership and study site will provide access to all trial-related sites, source data/documents, and reports for the purpose of monitoring and auditing by the sponsor and inspection by local and regulatory authorities.

### Ethical approval

This study was approved by the Ethics Review Committee, Faculty of Medicine, University of Ruhuna, Sri Lanka (reference no. 2023/P/113), on 30th November 2023 and the Duke University Institutional Review Board (IRB) (IRB no. Pro00114347) on 4th September 2024. Furthermore, this clinical trial was registered in ClinicalTrials.gov (ClinicalTrials.gov identifier: NCT06331364) and in the Sri Lanka Clinical Trial Registry (reference no. SLCTR/2024/019). Additionally, obtaining administrative clearance from the Education, Training, and Research (ET&R) Unit of the Ministry of Health, Sri Lanka, was mandatory. Any amendments to the protocol, other than administrative ones, will be approved by the University of Ruhuna ERC, Duke University IRB, and SLCTR according to their procedures.

### Trial status

The current protocol version is V7, 10.12.2025. This clinical trial is currently awaiting necessary regulatory approvals from DGM, BHB, and NHG to initiate the enrollments. It is anticipated that enrollments into the trial will commence in February 2026. Enrollment is expected to be completed in May 2027.

## Discussion

This stepped-wedge cluster randomized clinical trial will assess the impact of a novel eCDST, RespiQuestAB, on antibacterial use and clinical outcomes among patients hospitalized with LRTI in three public hospitals in Southern Sri Lanka. We expect that the results of this study will contribute to the strategic implementation of evidence-based diagnostic and treatment recommendations to improve the management of LRTI potentially throughout the country.

The extended data collection period (up to 2 years) makes the trial vulnerable to unexpected systemic changes beyond our control. Poor enrollment, malfunctioning or technical issues of the mobile application during the intervention, poor engagement of medical professionals especially during busy hours or admission days, and difficulties in conducting tests recommended by the mobile application could be potential challenges for TREAT-SL in achieving the expected outcomes. There is also the possibility that the treatment recommendations may result in worse clinical outcomes if antibacterials are withheld unnecessarily; however, monitoring by the DSMB will help reduce the negative impact of such an outcome. We expect that the implementation of RespiQuestAB, which is compliant with local regulations, could potentially improve clinical care in Southern Sri Lanka.

### Dissemination

The outcomes of this clinical trial will be published in peer-reviewed medical or public health journals with open access to ensure accessibility to a wide range of the research community, especially in LMICs. Manuscripts will also be submitted for digital archiving in PubMed Central upon acceptance for publication. Additionally, results will be presented at local and international conferences and will also be shared with the clinicians and hospitals involved in the trial.

The full protocol will be provided as a supplementary document upon publication of the results. We have not received permission from the regulatory authorities to share participant-level datasets. However, we may be able to publish the statistical code as supplementary documentation upon publication of the results.

## Supplementary Information

Additional file 1. Data Safety Monitoring Board Charter document of TREAT-SL. This includes the charter document of the DSMB established for the TREAT-SL clinical trial

Additional file 2. SPIRIT checklist. Additional file 2 includes the completed SPIRIT checklist according to the SPIRIT reporting guidelines

## Data Availability

No datasets were generated or analyzed during the current stage of the study. Data will be made available upon reasonable request once participant recruitment and data collection are complete, in accordance with ethical and institutional guidelines.

## Abbreviations

AE(s): Adverse event(s)
AFI: Acute febrile illness
ALMANACH: Algorithm for the management of childhood illness
BHB: Base Hospital Balapitiya
BiPAP: Bilevel positive airway pressure
CC: Coordinating center
COVID-19: Coronavirus disease 2019
CPAP: Continuous positive airway pressure
CRP: C-reactive protein
CT: Computerized tomography (scan)
DCRI: Duke Clinical Research Institute
DGM: District General Hospital Matara
DSMB: Data and Safety Monitoring Board
DOOR: Desirability of Outcome Ranking
eCDST: Electronic Clinical Decision Support Tool
ERC: Ethical Review Committee
ESR: Erythrocyte sedimentation rate
GEE: Generalized estimating equations
HIV: Human immunodeficiency virus
hMPV: Human metapneumovirus
HRV/HEV: Human rhinovirus/enterovirus
ICC: Intracluster correlation coefficient
ICF: Informed consent form
ICU: Intensive care unit
IMAI: Integrated Management of Adolescent and Adult Illness
IMCI: Integrated Management of Childhood Illness
IRB: Institutional Review Board
LAR: Legally authorized representative
LMIC(s): Low- or middle-income country/countries
LRTI: Lower respiratory tract infection
NIH: The National Institutes of Health
NHG: National Hospital Galle
NP: Nasopharyngeal
PCT: Procalcitonin
POCT: Point-of-care test
PI(s): Principal investigator(s)
POC: Point of care
RSV: Respiratory syncytial virus
SAE(s): Serious adverse event(s)
TREAT-SL: TREATment of Lower Respiratory Tract Infection in Selected Hospitals in Southern Sri Lanka
UAP: Unanticipated problem
WHO: World Health Organization
