# Supplementary material for: TREATment of Lower Respiratory Tract Infection in Selected Hospitals in Southern Sri Lanka (TREAT-SL): study protocol for a stepped-wedge, cluster-randomized clinical trial
Source: Trials. 2026 Mar 18;27:324. doi: 10.1186/s13063-026-09628-0 (PMC13113038; doi:10.1186/s13063-026-09628-0)
Supplement: Supplementary file 1 — Additional file 1. Data Safety Monitoring Board Charter document of TREAT-SL. This includes the charter document of the DSMB established for the TREAT-SL clinical trial [file 13063_2026_9628_MOESM1_ESM.pdf]

Data Safety Monitoring Board Charter

TREATment of Lower Respiratory Tract Infection in Sri Lanka (TREAT-SL):
A Stepped-wedge Cluster Randomized Trial of an Electronic Clinical Decision Support Tool (eCDST) for the Diagnosis and Treatment of Lower Respiratory Tract Infection (LRTI) in Southern Sri Lanka

Version 2.0

**Study Sponsor**	National Institute of Allergy and Infectious Diseases (NIAID)
**Protocol Number**	Pro00114347

Prepared by: Stefany Olague, MPH, DCRI Project Leader

Approval page

Table of Contents .... Page

1. ABBREVIATIONS 2

2. INTRODUCTION 3

3. PRIMARY ROLE OF THE DSMB 3

4. COMPOSITION OF THE DSMB 3

5. RESPONSIBILITIES OF THE DSMB 5

6. DSMB MEETINGS 5

6.1 Meeting Schedule 5
6.2 Meeting Format 5
6.2.1 Open Session 6
6.2.2 Closed Session 6
6.2.3 Executive Session 6
6.3 Reports to the DSMB 7
6.3.1 Open Session Report 7
6.3.2 Closed Session Report 8
6.4 DSMB Recommendation 8
6.5 DSMB Formal Summary 8
6.6 Procedures for Recommendations to the Steering Committee 9

7. Conflict of Interest Guidelines 9

APPENDIX A: DSMB Formal Summary 10

Appendix B: DSMB Recommendation Form 11

Appendix C: DSMB member contact information 12

Appendix D: Key Study Contacts 13

1. ABBREVIATIONS

Abbreviation	Description
**AE**	Adverse Event
**CFR**	Code of Federal Regulations
**DCRI**	Duke Clinical Research Institute
**DSMB**	Data Safety Monitoring Board
**eCDST**	Electronic Clinical Decision Support Tool
**LRTI**	Lower Respiratory Tract Infection
**NIAID**	National Institute of Allergy and Infectious Diseases
**PI**	Principal Investigator
**PL**	Project Lead
**SAE**	Serious Adverse Event
**SC**	Steering Committee
**TREAT-SL**	TREATment of Lower Respiratory Tract Infection in Sri Lanka

2. INTRODUCTION

This document is the Charter of the Data Safety Monitoring Board (DSMB) for the TREATment of Lower Respiratory Tract Infection in Sri Lanka (TREAT-SL): A Stepped-wedge Cluster Randomized Trial of an Electronic Clinical Decision Support Tool (eCDST) for the Diagnosis and Treatment of Lower Respiratory Tract Infection (LRTI) in Southern Sri Lanka.

The primary objective of the study is to determine the impact of an electronic clinical decision support tool (eCDST) on clinical outcomes and antibacterial prescription in subjects with LRTI in the intervention group compared to the control group.

This study is a stepped-wedge, cluster-randomized, two-arm, open-label, clinical trial of an electronic clinical decision support tool (eCDST) for the diagnosis and treatment of lower respiratory tract infection (LRTI) among patients at three sites in Southern Province, Sri Lanka.

The study will enroll 765 patients ≥ 14 years of age. Medical wards will be randomized in clusters to the intervention at intervals of 3-6 months until all clusters cross over. Participants will be followed for 30 days from enrollment to record clinical outcomes and any antimicrobials prescribed.

3. PRIMARY ROLE OF THE DSMB

The DSMB is advisory to the clinical trial leadership group, hereafter referred to as the Steering Committee (SC).

The DSMB is primarily responsible for protecting trial participants from harm due to the study intervention or as a result of undergoing study procedures, and for monitoring the overall conduct of the trial. The DSMB periodically reviews accumulating trial data related to safety and makes a benefit-risk assessment. After each meeting where data is reviewed, the DSMB will make a recommendation to continue the study as is, continue with modification, or terminate.

4. COMPOSITION OF THE DSMB

The DSMB is an independent multidisciplinary group that collectively has experience in the management of adult patient populations, lower respiratory tract infections, administration of antimicrobials, and monitoring of randomized clinical trials. The affiliations and expertise of the DSMB members are listed below. Contact information for DSMB members and key study contacts are listed in Appendix C and Appendix D, respectively.

The DSMB Chair and members are selected by the SC in consultation with the Sponsor. The DSMB chair is responsible for leading all meetings, facilitating effective interactions in the group, ensuring completion of timely and accurate minutes, and communicating recommendations to clinical trial leadership. The members are expected to attend all DSMB meetings to the best of their abilities and to provide expert opinions in group discussions.

DSMB Member Affiliations and Areas of Expertise

**Official DSMB members**		
Full Name, Credentials	Affiliation	Area(s) of Expertise
Neil Stafford (Chair)	Duke University School of Medicine	General internal medicine, acute respiratory tract infections, antimicrobial prescription
Cynthia Coffman	Duke University School of Medicine	Biostatistics, epidemiology
Shehan De Silva	Faculty of Medicine, University of Sri Jayawardenapura, Sri Lanka	General internal medicine, acute respiratory tract infections, antimicrobial prescription
**Duke Statistics Team (Blinded)**		
Hrishikesh Chakraborty	Duke Clinical Research Institute	Biostatistics, clinical trials, epidemiology
Obale Armstrong	Duke Global Health Institute	Biostatistics, epidemiology
Bhagya Senadheera	Ruhuna-Duke Centre for Infectious Diseases, Faculty of Medicine, University of Ruhuna, Sri Lanka	Biostatistics, epidemiology, bioinformatics
**Duke Statistics Team (Unblinded)**		
Avi Kenny	Duke Global Health Institute	Biostatistics, clinical trials, epidemiology
John Gallis	Duke Global Health Institute	Biostatistics, epidemiology, bioinformatics
Kate Sanborn	Duke Global Health Institute	Biostatistics, epidemiology

5. RESPONSIBILITIES OF THE DSMB

To fulfill its role in trial oversight the DSMB will do the following:
- Review the study protocol and amendment(s) and, when appropriate, provide comments to the SC.
- Review overall data collection methods and safety monitoring procedures, recommending modifications as needed
- Evaluate the progress of the trial, including periodic assessments of data quality and timeliness, participant recruitment, accrual and retention, participant risk versus benefit, trial site performance, and other factors that can affect study outcome
- Consider factors external to the study when relevant information becomes available, such as scientific or therapeutic developments that may have an impact on participant safety or the ethics of the trial
- Review study performance, make recommendations and assist in the resolution of problems reported by the Principal Investigators
- Protect the safety of the study participants
- DSMB Reporting and Meetings

6. DSMB MEETINGS

6.1 Meeting Schedule

The DSMB meeting will be scheduled based on availability of required attendees. The DSMB meeting will be held as a teleconference. If required members of the DSMB are not available at the time of the scheduled safety review meeting, they must send written feedback on the safety data. If members of the DSMB team are not available for the DSMB teleconference and cannot provide written feedback, a teleconference with at least all required DSMB members in attendance must be convened to discuss the data.

The first DSMB meeting will take place prior to study initiation to review the study protocol, informed consent form, data, safety and monitoring plans, and the DSMB charter. Subsequent meetings will be scheduled every 6 months or at the DSMB's request.

6.2 Meeting Format

Members of the DSMB team will receive a meeting agenda and the safety data from the study PI or designee for review at least 2 weeks prior to a scheduled DSMB meeting.

Meetings shall be closed to the public because discussions may address confidential patient data. Meetings may be convened as conference calls or webinars, as well as in person. In special circumstances, the meetings may also be conducted by email. An

emergency meeting of the DSMB may be called at any time by the DSMB chairperson should questions of patient safety arise.

The DSMB Chair will lead the DSMB meetings. A summary of the data will be presented. There will be an opportunity for open discussion of the data and overall study conduct. At the conclusion of the discussion, the DSMB team votes on whether to proceed with the study as outlined in the protocol.

Results of the DSMB meeting will be documented in meeting minutes and sent for review and approval by the DSMB team members. The DSMB meeting minutes will be completed by the DSMB Chair and, once approved, may be shared with the study team. Appendix A outlines the format for meeting minutes.

6.2.1 Open Session

Members of the DSMB, the principal investigator and members of the steering committee, including the study biostatisticians may attend the open session. Issues discussed will include the conduct and progress of the study, including patient recruitment, data quality, general adherence, adverse events, compliance with protocol, and any other logistical matters that may affect either the conduct or outcome of the study. Proposed protocol amendments will also be presented in this session. Treatment group data may not be presented in the open session.

6.2.2 Closed Session

The closed session will be attended only by DSMB members, and the unmasked study biostatisticians. The discussion at the closed session is completely confidential. All materials from the closed session will be destroyed at the end of the meeting.

Because of differing recruitment by intervention arm over the course of the study, it will not be possible to mask the summaries by arm. Analyses of outcome data are reviewed by intervention groups, including baseline characteristics, primary and key secondary outcomes, adverse events, adherence and dropouts, and examination of any relevant subgroups.

6.2.3 Executive Session

The executive session will be attended by DSMB members only, who will discuss the information presented during the closed and open sessions and provide input on the continuation or termination of the study, protocol modification or other changes to the conduct of the study.

The DSMB will make a recommendation for either continuation or termination of the study. Termination may be suggested by the DSMB at any time. Reasons for early termination include:

- Serious adverse effects in entire intervention group or in a dominating subgroup;
- Logistical or data quality problems so severe that correction is not feasible.

Sound rationale for either decision (continuation or termination of the study) should be presented.

6.3 Reports to the DSMB

Data for the DSMB report will be obtained directly from the REDCap database. Refer to the DSMB statistical analysis plan (SAP) for table shells outlining the expected presentation of the data.

The following data types will be included in the safety review process:

- A summary of the study status, including the target enrollment, current and projected time to completing enrollment. Any significant events and/or difficulties will be briefly described in the summary
- A summary describing gender, age, ethnicity and other relevant demographic characteristics
- A summary describing participants' completion of all study activities (*i.e.*, completion of 30-day follow up visit)
- A summary of AEs and SAEs
- A listing of AE details grouped by participant
- A listing of SAE details grouped by participant
- A listing of deaths
- A listing of protocol deviations

6.3.1 Open Session Report

This portion of the report provides information on study aspects such as accrual, baseline characteristics, and other general information on study status. This report is generally shared with all investigators involved with the clinical study. The reports contained in this section generally include:

- Comparison of Target Enrollment to Actual Enrollment by Month;
- Comparison of Target Enrollment to Actual Enrollment by Site;
- Comparison of Target Enrollment to Actual Enrollment by Cluster;
- Overall Subject Status by Site, including: Subjects Screened, Enrolled, Active, Completed and Terminated;
- Demographic and Key Baseline Characteristics by Group;
- Adverse Events/Serious Adverse Events by Site and Subject

6.3.2 Closed Session Report

This report may contain data on study outcomes related to safety as well as other safety measures. The Closed Session Report is considered confidential and should be destroyed at the conclusion of the meeting. Data files to be used for analyses should have undergone established editing procedures to the extent possible. This report should not be viewed by any members of the clinical study except the designated unmasked study statisticians.

6.4 DSMB Recommendation

Efforts should be made for all DSMB members to attend each meeting, whether scheduled or unscheduled. Each meeting must include one or more representatives from the Duke Statistical Teams (blinded and unblinded).

Consensus on the DSMB recommendation is desired. If the DSMB is unable to achieve consensus on a proposal, motion, or recommendation, the Chair may elect to conduct a vote. If a decision is made without consensus, dissenting views should be clearly described in the meeting minutes and in the recommendation to the SC.

At the discretion of the Chair, absent members may be contacted before or after a meeting to discuss and obtain their views on proposed recommendations. If recommendations involve major changes to the study, or if the meeting did not have a quorum, the Chair should arrange for a follow-up meeting or teleconference including the full DSMB.

6.5 DSMB Formal Summary

A formal summary containing the DSMB’s input on the conduct of the study and their recommendation regarding continuation of the study will be prepared by the DSMB Chairperson. Each DSMB summary will include the DSMB’s recommendation regarding continuation or termination of the study. Refer to Appendix A for a template of the formal summary.

The DSMB meeting summary will not include unmasked data, discussion of the unmasked data, or any other confidential data. Once completed, the summary is sent to the DSMB members for their review and concurrence. When the summary is satisfactory to the DSMB members and concurrence with the summary is received, the summary will be sent to the PI within 7 days of the DSMB meeting. It is the responsibility of the PI to distribute the summary to all co-investigators. It is the responsibility of the study investigators to assure that the DSMB summary is submitted to all the Institutional Review Boards (IRBs) associated with the study.

The formal summary should include a statement that each member in attendance was reminded to disclose any conflicts of interest.

6.6 Procedures for Recommendations to the Steering Committee

The DSMB Chair will provide its recommendations to the study leadership and SC simultaneously via email within 3 days of the conclusion of the DSMB meeting. Refer to Appendix B for the recommendation form template.

The study leadership may meet with the DSMB to gain further understanding of the recommendation. Attempts should be made to resolve any disagreements among the DSMB and SC through discussion or presentation of additional data analysis. The formal summary will record any decisions/agreements that are made and will identify any additional data that has been disclosed.

7. Conflict of Interest Guidelines

The DSMB membership is restricted to individuals free of significant conflicts of interest. The source of these conflicts many be financial, scientific or regulatory in nature.

Individuals invited to serve on the DSMB as either voting or non-voting members must disclose any potential conflicts of interest, whether real or perceived. Conflicts of interest can include professional, proprietary, and miscellaneous interests as described in the NIH Grant Policy Statement and 45 CFR Part 94. Potential conflicts that develop during a member's tenure on a DSMB must also be disclosed. Written documentation attesting to an absence of conflict of interest is required annually.

Any member who develops a potential conflict of interest during the course of the trial should inform other members of the DSMB. If the potential conflict of interest is determined to be substantial and relevant, the member should resign from the DSMB.

APPENDIX A: DSMB Formal Summary

The formal summary will include

1. Full name of attendees
2. Date and time of meeting, and whether it is face-to-face vs. teleconference
3. Decisions, feedback, and action items
4. Reference to the DSMB rather than its individual members in the main body of minutes
5. Conflict of interest statement from each member in attendance

Appendix B: DSMB Recommendation Form

Study Name: TREAT-SL
Study Intervention: eCDST for LRTI management

Recommendation Form

The purpose of this form is to inform study investigators of the scope and outcome of the TREAT-SL DSMB deliberations on [insert DSMB meeting date].

Meeting date:
Database cut-off date:

After review of all available data, the DSMB makes the following recommendation:
- ☐ Continuation of study under the current study protocol
- ☐ Continuation of study with recommended changes to the protocol
- ☐ Discontinuation of the study

List of documents reviewed by the DSMB

DSMB Findings and Recommendations

Next Meeting
The DSMB will conduct an electronic review of safety data report when it's available. The next regularly scheduled DSMB meeting will be in 6 months (insert estimated month year).
Additional Comments
……
……
……
…

Signature

xxxxxx, Chair
On behalf of the DSMB Members

Appendix C: DSMB Members Contact Information

Name	E-mail
**Neil K. Stafford, MD**	neil.stafford@duke.edu
**Cynthia Coffman, PhD**	cynthia.coffman@duke.edu
**Shehan De Silva**	dshehans@gmail.com

Appendix D: Key Study Contacts

Name	StudyRole	E-mail
**Gayani Tillekeratne, MD MSc**	PI	gayani.tillekeratne@duke.edu
**Warsha De Soyza, MBBS MD**	Local PI	warshadez@gmail.com
**Champica Bodinayake, MBBS MD**	Co-I	bodinayake@gmail.com
**Gaya Wijayaratne, MBBS MD**	Co-I	gayabw@yahoo.co.uk
**Christopher Woods, MD MPH**	Co-I	chris.woods@duke.edu
**Susanna Naggie, MD MHS**	Co-I	susanna.naggie@duke.edu
**Hrishikesh Chakraborty, PhD**	Co-I	hrishikesh.chakraborty@duke.edu
**Maria Iglesias De Ussel, PhD**	Research Scientist	maria.iglesiasdeussel@duke.edu
**Avi Kenney, PhD**	Biostatistician	avi.kenny@duke.edu
**Bhagya Senadheera, PhD**	Bioinformatician	bhagya.senadheera@duke.edu
**John Gallis, MS**	Biostatistician	john.gallis@duke.edu
**Kate Sanborn, MS**	Biostatistician	kate.sanborn@duke.edu
**Chrissy Nix**	Database manager	christina.nix@duke.edu
**Obale Armstrong, MS**	Research/Data Analyst	christina.nix@duke.edu
**Jayani Gamage**	Study coordinator	jayanigamage03@gmail.com
**Stefany Olague, MPH**	PL	stefany.olague@duke.edu
