# Supplementary material for: TREATment of Lower Respiratory Tract Infection in Selected Hospitals in Southern Sri Lanka (TREAT-SL): study protocol for a stepped-wedge, cluster-randomized clinical trial
Source: Trials. 2026 Mar 18;27:324. doi: 10.1186/s13063-026-09628-0 (PMC13113038; doi:10.1186/s13063-026-09628-0)
Supplement: Supplementary file 2 — Additional file 2. SPIRIT checklist. Additional file 2 includes the completed SPIRIT checklist according to the SPIRIT reporting guidelines [file 13063_2026_9628_MOESM2_ESM.docx]

# **SPIRIT Checklist for *Trials***

Complete this checklist by entering the page and line numbers where each of the items listed below can be found in your manuscript.

Your manuscript may not currently address all the items on the checklist. Please modify your text to include the missing information. If you are certain that an item does not apply, please state "n/a" and provide a short explanation. **Leaving an item blank or stating “n/a” without an explanation will lead to your manuscript being returned before review.**

Upload your completed checklist as an additional file when you submit to *Trials*. You must reference this additional file in the main text of your protocol submission. The completed SPIRIT figure must be included within the main body of the protocol text and can be downloaded here: <http://www.spirit-statement.org/schedule-of-enrolment-interventions-and-assessments/>

In your methods section, please state that you used the SPIRIT reporting guidelines, and cite them as:

Chan A-W, Tetzlaff JM, Gøtzsche PC, Altman DG, Mann H, Berlin J, Dickersin K, Hróbjartsson A, Schulz KF, Parulekar WR, Krleža-Jerić K, Laupacis A, Moher D. SPIRIT 2013 Explanation and Elaboration: Guidance for protocols of clinical trials. BMJ. 2013;346:e7586

|  |  | **Reporting Item** | **Page and Line Number** | **Reason if not applicable** |
| --- | --- | --- | --- | --- |
| **Administrative information** | | | | |
| Title | [#1](https://www.goodreports.org/reporting-checklists/spirit/info/#1) | Descriptive title identifying the study design, population, interventions, and, if applicable, trial acronym | Page 1, Line 1-3 | **TREATment of Lower Respiratory Tract Infection in Selected Hospitals in Southern Sri Lanka (TREAT-SL): Study Protocol for a Stepped-Wedge Cluster-Randomized Clinical Trial** |
| Trial registration | [#2a](https://www.goodreports.org/reporting-checklists/spirit/info/#2a) | Trial identifier and registry name. If not yet registered, name of intended registry | Page 4, Line 116-117 and page 28, Line 620-621 | ClinicalTrials.gov Identifier: NCT06331364  Sri Lanka Clinical Trial Registry (SLCTR) registration no: SLCTR/2024/019 |
| Trial registration: data set | [#2b](https://www.goodreports.org/reporting-checklists/spirit/info/#2b) | All items from the World Health Organization Trial Registration Data Set |  | WHO ICTRP registration, Main ID NCT06331364 |
| Protocol version | [#3](https://www.goodreports.org/reporting-checklists/spirit/info/#3) | Date and version identifier | Page 28, Line 627 | V7 (10-12-2025) |
| Funding | [#4](https://www.goodreports.org/reporting-checklists/spirit/info/#4) | Sources and types of financial, material, and other support | Page 32, Line 678-679 | The TREAT-SL trial was funded by the National Institute of Allergy and Infectious Diseases, USA (grant no: R01AI168420) |
| Roles and responsibilities: contributorship | [#5a](https://www.goodreports.org/reporting-checklists/spirit/info/#5a) | Names, affiliations, and roles of protocol contributors | Page 33 | Principal Investigators: Gayani Tillekeratne, MD MSc  Duke University School of Medicine  Warsha De Zoysa, MBBS MD  Faculty of Medicine, University of Ruhuna  Co-Investigators: Champica Bodinayake, MBBS MD  Faculty of Medicine, University of Ruhuna  Gaya Wijayaratne, MBBS MD  Faculty of Medicine, University of Ruhuna  Christopher Woods, MD MPH  Duke University School of Medicine  Susanna Naggie, MD MHS  Duke University School of Medicine  **Hrishikesh Chakraborty, PhD**  Duke University School of Medicine |
| Roles and responsibilities: sponsor contact information | [#5b](https://www.goodreports.org/reporting-checklists/spirit/info/#5b) | Name and contact information for the trial sponsor | Page 32, Line 679 | National Institute of Allergy and Infectious Diseases, Maryland, USA. Contact no: [+1 301-496-2263](https://www.google.com/search?q=National+Institute+of+Allergy+and+Infectious+Diseases%2C+Maryland%2C+USA+contact+info&rlz=1C1UEAD_enLK1186LK1189&oq=National+Institute+of+Allergy+and+Infectious+Diseases%2C+Maryland%2C+USA+contact+info&gs_lcrp=EgZjaHJvbWUyBggAEEUYOdIBCjEyMDMwajBqMTWoAgiwAgHxBWcePfK8Cqxg&sourceid=chrome&ie=UTF-8) |
| Roles and responsibilities: sponsor and funder | [#5c](https://www.goodreports.org/reporting-checklists/spirit/info/#5c) | Role of study sponsor and funders, if any, in study design; collection, management, analysis, and interpretation of data; writing of the report; and the decision to submit the report for publication, including whether they will have ultimate authority over any of these activities | Page 32,Line 679-682 | Sponsor is noncommercial and declares no conflict of interest. The sponsor approved the study design. The sponsor played no part in study design; collection, management, analysis, and interpretation of data; the writing of the report; and the decision to submit the report for publication. |
| Roles and responsibilities: committees | [#5d](https://www.goodreports.org/reporting-checklists/spirit/info/#5d) | Composition, roles, and responsibilities of the coordinating centre, steering committee, endpoint adjudication committee, data management team, and other individuals or groups overseeing the trial, if applicable (see Item 21a for data monitoring committee) | Pages 25,26, Line 560- 578 | The Coordinating Center (CC) will be at the Duke Clinical Research Institute (DCRI). The CC functions as a clinical trial center and is responsible for project management, oversight of all committees and working groups in the US; development of the protocol and all amendments; quality control; monitoring of study progress; and leadership in data analysis, presentations, and publications.  Trial oversight will be conducted by members of the core research team. This steering committee is comprised of investigators from both the USA and Sri Lanka, including physicians, the blinded statistical team, and research coordinators/ staff. This steering committee will meet bi-weekly via zoom to review trial progress, recruitment status, protocol adherence, and data quality issues. Prior to the steering committee meetings, data for key variables related to study monitoring and progress will be collated using an R script that runs on data inputted into the REDCap database. These data will be presented and discussed during the steering committee meetings.  Day-to-day trial operations are managed by a local project coordinator, who is responsible for regulatory compliance, coordination and supervision of research assistants, monitoring participant recruitment, and providing regular updates to the principal investigators and other team members. The site PI will oversee staff training, ensure adherence to the study protocol, and oversee ongoing monitoring of study activities. |
| **Introduction** |  |  |  |  |
| Background and rationale | [#6a](https://www.goodreports.org/reporting-checklists/spirit/info/#6a) | Description of research question and justification for undertaking the trial, including summary of relevant studies (published and unpublished) examining benefits and harms for each intervention | Page 4-8, Line 121-204 | Globally, lower respiratory tract infection (LRTI) is among the leading causes of hospitalization and is a major driver of antibacterial use and overuse (1)(2). Viral and bacterial LRTI have similar presentations, leading clinicians to overprescribe antibacterials for fear of missing an otherwise lethal bacterial infection or superinfection (3). Antibacterial overuse is worse in low- and middle-income countries (LMICs), where LRTI remains the leading infectious cause of mortality and where diagnostic capacity is limited (1),(4–6). However, emerging data from both pediatric and adult cohorts indicate that viral LRTIs are often recognized more commonly than bacterial LRTIs. In many countries, respiratory viruses are commonly observed to cause LRTIs in both children and adults (4),(7–9). In Sri Lanka, our team has shown that respiratory viruses can be identified in almost 40% of patients hospitalized with LRTI, with the most commonly detected viruses being influenza A, human rhinovirus/enterovirus (HRV/HEV), and respiratory syncytial virus (RSV). In this previous study, 84.4% of children and 87.8% of adult patients with a respiratory virus identified were treated with antibiotics during hospitalization (10). We have also shown that LRTI is the most common indication for antibiotic use in the inpatient setting in five public hospitals in Southern Province, Sri Lanka (11).The unnecessary use of antibacterials for treatment of viral infection increases downstream antimicrobial resistance, which is estimated to cause up to 10 million deaths annually by 2050 (12). Conversely, lack of antibactieral use for bacterial LRTI is associated with increased morbidity and mortality (13). Thus, optimally targeting antibacterials, with antibacterial prescription for bacterial LRTI and antibacterial avoidance for viral LRTI, is vital in the care of patients with LRTI.  Access to diagnostic test results can reduce inappropriate antibacterial use for respiratory viral infection and help target therapy (14–17). In some countries, diagnostic tests have been shown to significantly reduce antibacterial prescriptions for viral respiratory infections (17,18). In Sri Lanka, our team has shown that a positive rapid influenza test was associated with a 20% reduction in antibacterial prescriptions (84% versus 62%) among patients with influenza (18). However, limited availability of diagnostics continues to drive inappropriate antimicrobial use for LRTIs (3)(19). Pathogen-based tests, like sputum or blood cultures and multiplex polymerase chain reaction (PCR) of nasopharyngeal samples, may have limited sensitivity, cover few organisms, and fail to distinguish colonization from infection (19). Additionally, detection of a pathogen from the upper respiratory tract may not indicate the etiology of infection in the lower respiratory tract (3). In LMICs, the use of existing diagnostics is further hampered by lack of access and high cost (20)(21). Given limitations associated with pathogen-based tests, host-based diagnostics, which broadly classify infections as bacterial or viral, may provide valuable adjunctive information in diagnosing and managing antimicrobials for respiratory infection (22)(23). Host biomarkers act as surrogate measures of the immune response to infection and do not depend on identifying a specific pathogen (24)(25). C-reactive protein (CRP) and procalcitonin (PCT) are the most widely studied biomarkers to date for classifying viral versus bacterial respiratory infection (26)(27)  Evidence-based algorithms for the diagnosis and treatment of LRTI are not widely available in high-income or LMIC settings. Especially in LMICs, where access to diagnostics may be limited, strategies based on local epidemiology are critical for maximizing impact and minimizing cost. For LMICs, the Integrated Management of Childhood Illness (IMCI) and Integrated Management of Adolescent and Adult Illness (IMAI) developed by the World Health Organization (WHO) provide basic strategies on diagnosing and treating conditions such as LRTI (28)(29)(30). These algorithms rely on syndromic definitions that can be applied broadly in resource-limited settings, and have been associated with better health-worker performance and quality of care (30). However, the IMCI/IMAI do not capture local epidemiology of infection (31). In addition, these guidelines were designed to maximize sensitivity for bacterial infection over specificity, with the result that more patients than necessary may receive antibacterials (32). New strategies for diagnosing acute febrile illness (AFI) in resource-limited settings are starting to be explored. Pokharel *et al.* demonstrated serial point-of-care (POC) testing for AFI causes may be more accurate than simultaneous testing in India and Cambodia (33). A meta-analysis of three randomized clinical trials conducted in Burkina Faso, Ghana and Uganda among patients with acute febrile illness showed that a diagnostic algorithm combined with POC tests (Malaria Rapid Diagnostic Test (RDT), CRP, White Blood Cell (WBC) total count and differential counts) could possibly reduce inappropriate antibacterial prescription without impairing patient outcomes (34). In Afghanistan and Nigeria, ALMANACH (Algorithm for the Management of Acute Childhood Illness), a digital IMCI tool, reduced antibiotic prescriptions for children (35). Keitel *et al.* developed a novel electronic algorithm (e-POCT) including POC tests (pulse oximetry, glucometer, hemoglobin, malaria test, rapid HIV, CRP, and PCT) to triage children <5 years with AFI; e-POCT-resulted in non-inferior clinical outcomes and decreased antibacterial use compared to ALMANACH (36).These strategies all emphasize the need for evidence-based algorithms, as etiology of infection as well as performance of biomarkers may vary based on local context (22).  Rationale  Duke University and Ruhuna University have had a formal research collaboration (Duke- Ruhuna Collaborative Research Center) since 2006. Multiple large cohort studies in infectious disease epidemiology have been conducted in the Southern Province, Sri Lanka, through this collaboration. Over the past decade, our study team has demonstrated that LRTI is the most common reason for antibacterial use in the inpatient setting in Southern Sri Lanka (11). In addition, we have shown that viruses such as influenza, RSV, and adenovirus are more commonly identified than bacteria as a cause of LRTI in Southern Sri Lanka (10).  In this study, we will leverage the rich body of epidemiologic information we have gathered in past years to develop and implement a tool to improve the diagnosis and management of LRTI, using Southern Sri Lanka as our initial test region. By conducting this interventional trial in the same setting as that in which we generated the initial data, we hope to produce a tool that is evidence based, locally relevant, culturally appropriate, and acceptable to local physicians.  The TREATment of Lower Respiratory Tract Infection in Sri Lanka (TREAT-SL) trial will evaluate the impact of a novel electronic clinical decision support tool (eCDST), known as RespiQuestAB, for managing LRTI. The rationale for the trial is that an evidence-based tool consisting of real-time surveillance data, clinical predictors, POC pathogen tests, and POC biomarker tests will result in improved management of LRTI.  Our hypothesis is that the use of the eCDST will reduce antibacterial prescription and result in non-inferior clinical outcomes. |
| Background and rationale: choice of comparators | [#6b](https://www.goodreports.org/reporting-checklists/spirit/info/#6b) | Explanation for choice of comparators | Page 15, Line 362-370 | There will be one interventional arm, which will include the use of the eCDST known as RespiQuestAB for diagnosing and managing LRTI. The RespiQuestAB will consist of an electronic application that physicians will be asked to download onto their smart phones. The eCDST uses machine learning and takes into account real-time incidence of influenza in Sri Lanka (updated weekly by study team), as well as epidemiological and clinical variables that are predictive of LRTI etiology, based on a biorepository of LRTI data we previously created in Sri Lanka.  The comparator will be standard for care, in order to assess the impact of the eCDST on antibacterial use and clinical outcomes. |
| Objectives | [#7](https://www.goodreports.org/reporting-checklists/spirit/info/#7) | Specific objectives or hypotheses | Pages 8-9, Line 207-219 | The primary objective of this study is to determine the impact of the RespiQuestAB on clinical outcomes and antibacterial prescription in subjects with LRTI in the intervention group compared to the control group. The secondary objectives include the following: to compare clinical outcomes at discharge and by Day 30 in the intervention and control groups; to compare the use of and duration of use of advanced care and therapies in the intervention and control groups; to compare the duration of hospitalization in the intervention and control groups; to compare antibacterial, oseltamivir, and SARS-CoV-2 antiviral prescription and duration of prescription between the intervention and control groups; and to determine physicians’ adherence to RespiQuestAB diagnostic test and treatment recommendations. The exploratory aims are to compare ordinal clinical outcomes by Day 30 in the intervention and control groups when using a Desirability of Outcome Ranking (DOOR) scale, and to compare SARS-CoV-2 immunomodulator prescription and duration of prescription between the intervention and control groups. |
| Trial design | [#8](https://www.goodreports.org/reporting-checklists/spirit/info/#8) | Description of trial design including type of trial (eg, parallel group, crossover, factorial, single group), allocation ratio, and framework (eg, superiority, equivalence, non-inferiority, exploratory) | Page 9, Line 229- 238 | This study is an open-label, stepped-wedge, cluster-randomized trial. The stepped-wedge design was chosen to increase logistical feasibility, increase participation in the study (since all clusters will eventually receive the intervention), reduce contamination bias, and reduce the risk of a cluster dropping out of the study because they are not receiving the intervention. This trial includes 9 clusters (5 in NHG, 2 in DGM, and 2 in BHB), with three clusters randomized to intervention at each step/ time period until all clusters receive the intervention. All clusters (pairs of wards) will be observed for four time periods, each of which will last between 3-6 months, with the time period ending when the target sample size is reached for that period. Participant sampling will be done on a repeated cross-sectional basis. The study design is depicted in Figure 1. |
| **Methods: Participants, interventions, and outcomes** | | | | |
| Study setting | [#9](https://www.goodreports.org/reporting-checklists/spirit/info/#9) | Description of study settings (eg, community clinic, academic hospital) and list of countries where data will be collected. Reference to where list of study sites can be obtained | Page 9, Line 221-227 | TREAT-SL will be conducted in three public hospitals in the Southern Province of Sri Lanka: National Hospital Galle (NHG), District General Hospital Matara (DGM), and Base Hospital Balapitiya (BHB). NHG is an 1,800-bed hospital in Galle district and the largest tertiary care center in the Southern Province. The hospital is affiliated with the Faculty of Medicine, University of Ruhuna. DGM is the largest hospital in Matara district, Sri Lanka, with approximately 1000 beds. BHB is the largest base (secondary) hospital in Galle District, Sri Lanka, and has approximately 500 beds. |
| Eligibility criteria | [#10](https://www.goodreports.org/reporting-checklists/spirit/info/#10) | Inclusion and exclusion criteria for participants. If applicable, eligibility criteria for study centres and individuals who will perform the interventions (eg, surgeons, psychotherapists) | Page 10-12, Line 244-292 | *Participant Inclusion Criteria*  In order to be eligible to participate in this study, an individual must meet all of the following criteria:   1. Admitted within prior 48 hours 2. Have evidence of new acute respiratory illness (<14 days of symptoms), as indicated by at least one of the following:    1. New cough or sputum production    2. Chest pain    3. Dyspnea or tachypnea (respiratory rate >20 breaths/minute)    4. Abnormal lung examination 3. Have evidence of acute infection, as indicated by at least one of the following:    1. Self-reported fever or chills    2. Documented fever ≥ 38 ̊C (100.4 ̊F)    3. Documented hypothermia < 35.5 ̊C (95.9 ̊F)    4. Leukocytosis (white blood cell count >10,000/ µL )    5. Leukopenia (white blood cell count < 3000/ µL)    6. New altered mental status 4. Ability and willingness of patient, parent or legally authorized representative (LAR) to give informed consent 5. Ability of children 14-17 years of age to provide assent 6. Ability to complete follow-up encounter at 30 days in person or by telephone   *Participant Exclusion Criteria*  Participants who meet any of the following criteria will be excluded from enrollment in this study:   1. Hospitalized recently (within last 28 days) 2. Enrolled into this clinical trial previously 3. Surgery in the past 7 days 4. Unable or unwilling to complete the follow-up encounter 5. Likely to be transferred from the medical wards within 24 hours of enrollment (to another ward or to another hospital) 6. Has underlying conditions or circumstances for which physicians would be unlikely to withhold antibacterials: 7. Vasopressor therapy 8. Cystic fibrosis 9. Known severe immunosuppression    - 1. Cancer or another condition with neutropenia (absolute neutrophil count <1000/ µL)      2. Solid-organ or hematopoietic stem-cell transplant within the previous 90 days      3. Active graft-versus-host disease or bronchiolitis obliterans      4. On chronic steroids equivalent to prednisone 20mg daily for ≥ 2 weeks or other targeted cytotoxic or biologic immunosuppressants within the prior 4 weeks      5. Human immunodeficiency virus infection with a CD4 cell count <200/ µL); 10. Has an accompanying non-respiratory infection 11. Has evidence of a lung abscess or empyema (7)(37) 12. Has respiratory failure at enrollment, evidenced by use of non-invasive or invasive ventilation |
| Interventions: description | [#11a](https://www.goodreports.org/reporting-checklists/spirit/info/#11a) | Interventions for each group with sufficient detail to allow replication, including how and when they will be administered | Pages 15-16, Line 362-392 | *Intervention:* The intervention will involve the use of the eCDST, known as RespiQuestAB, for diagnosing and managing LRTI. RespiQuestAB is an electronic application (depicted in Figure 3) that physicians will be asked to download onto their smart phones.  **Figure 3**: A) Initial screen of the electronic application, RespiQuestAB B) Home/ main screen C) Results and recommendations screen  RespiQuestAB implements a pre-trained machine learning algorithm and takes into account real-time incidence of influenza in Sri Lanka (updated weekly by study team), as well as epidemiological and clinical variables that are predictive of LRTI etiology, based on a biorepository of LRTI data we previously created in Sri Lanka. A maximum of 25 epidemiological/ clinical variables are included in the algorithm, including variables such as the presence of fever, number of days of cough, and white blood cell count. On average, clinicians will be asked to respond to approximately 10 of these questions about a patient’s presenting signs and symptoms. Based on the responses, RespiQuestAB may advise performing POC tests for influenza, SARS-CoV-2, *Streptococcus pneumoniae,* and/ or PCT, if a positive test result would increase the probability of the most likely diagnosis by at least 5%. Clinicians will also have the ability to perform these POC tests of their own accord, and will be able to see post-test probabilities based on the test result prior to performing the test. Given need for additional POC testing as per the sequential RespiQuestAB diagnostic section instructions, there may be an additional collection of a blood specimen (5mL maximum) and/ or up to 2 additional nasal or nasopharyngeal samples within 48 hours of enrollment. Based on inputs into RespiQuestAB, the tool will provide a probability of influenza, other viral infection (including COVID-19), bacterial infection, non-infectious condition, or indeterminate condition. Clinicians will then be provided with a treatment recommendation (treatment with antibacterials or oseltamivir, or withholding of these drugs) based on the most likely diagnosis. POC testing as advised by RespiQuestAB will be conducted by either clinical staff or trained research staff. If performed by research staff, results will be delivered on paper or electronically/ by telephone within 6 hours of testing to ≥2 primary clinicians on the team (including the consultant/ attending-level physician or senior registrar). Since NHG, DGM, and BHB do not have an electronic medical record system, clinicians will be asked to input the results from diagnostic testing and to follow the treatment recommendations as per the RespiQuestAB, but will be advised that the final decision regarding prescription of antimicrobials is entirely at their discretion. |
| Interventions: modifications | [#11b](https://www.goodreports.org/reporting-checklists/spirit/info/#11b) | Criteria for discontinuing or modifying allocated interventions for a given trial participant (eg, drug dose change in response to harms, participant request, or improving / worsening disease) | Page 17 Line 407-413 | During the intervention, use of the intervention may be discontinued for a given trial participant by the PI due to any clinical adverse event, laboratory abnormality, or other medical condition or situation if continued participation in the study would be detrimental to the participant. Participation may also be discontinued if the participant meets an exclusion criterion (either newly developed or not previously recognized) that precludes further study participation. Moreover, the study may be discontinued for a given participant upon participant/guardian request or by withdrawal of the informed consent. |
| Interventions: adherance | [#11c](https://www.goodreports.org/reporting-checklists/spirit/info/#11c) | Strategies to improve adherence to intervention protocols, and any procedures for monitoring adherence (eg, drug tablet return; laboratory tests) | Pages 16-17, Line 393-406 | The principal investigators (PIs), co-investigators, and research team members will be responsible for overseeing compliance with all study parameters from enrollment to discharge. After discharge, a designated research assistant will be in touch with the participant or guardian to ensure completion of the follow-up assessment on day 30.  There is a separate section in the questionnaire/ case report form (CRF) to monitor adherence to the use of the intervention, RespiQuestAB. This section, which will be completed by research assistants, includes all details required to assess adherence, such as diagnostic tests recommended by the eCDST, whether the recommended tests were performed, the test results, and the whether the treatment recommendations were followed. In addition, the treating physicians will respond to questions in the RespiQuestAB app after each case in indicate whether they conducted the recommended diagnostic tests and whether they followed the treatment recommendations.  Physician adherence to RespiQuest will be enhanced through strategies such as providing initial training via a workshop, minimizing data entry into the app (approximately 10 variables), providing compensation for internet access, and addressing barriers in real time by the site PI. |
| Interventions: concomitant care | [#11d](https://www.goodreports.org/reporting-checklists/spirit/info/#11d) | Relevant concomitant care and interventions that are permitted or prohibited during the trial | Page 15, Line 355-361 | *Usual care:* Usual care arm is permitted. Study staff will inform the treating clinicians to diagnose and treat patients according to usual practice. Clinicians will be able to order routine diagnostic testing as per standard practice. These tests may include complete blood count, chemistries, CRP testing, erythrocyte sedimentation rate (ESR) testing, blood and sputum cultures, and chest x-ray or chest CT imaging. Apart from the intervention, participants included in both arms will receive usual care as per the clinician’s prescription. Therefore, participants are allowed to receive any treatment or therapies prescribed by the clinician. |
| Outcomes | [#12](https://www.goodreports.org/reporting-checklists/spirit/info/#12) | Primary, secondary, and other outcomes, including the specific measurement variable (eg, systolic blood pressure), analysis metric (eg, change from baseline, final value, time to event), method of aggregation (eg, median, proportion), and time point for each outcome. Explanation of the clinical relevance of chosen efficacy and harm outcomes is strongly recommended | Pages 18-21, Line 418-469 and pages 38-40 | The primary outcome will consist of a co-primary endpoint: 1) total duration of antibacterial prescription for the index visit (superiority analysis), and 2) clinical outcomes by Day 30 (non-inferiority analysis). Total duration of antibacterial prescription for the index visit will include the number of days that antibacterials are prescribed during the index hospitalization, as well as the number of days that antibacterials are prescribed at discharge from the hospital (intended use), for a maximum total of 14 days. The time of assessment will be 30 days (after enrollment) or at discharge. A day of antibacterial prescription will be defined as each day a subject is prescribed any oral, intramuscular, or intravenous antibacterial (may be non-consecutive days), excluding anti-virals, anti-fungals, or anti-parasitics. The primary, secondary and exploratory outcomes of the TREAT-SL trial are listed in Table 1.   \| **Table 1**: Primary, secondary and exploratory outcomes of the TREAT-SL trial \| \| \| \| --- \| --- \| --- \| \| **Category** \| **Outcome** \| **Details** \| \| **Primary outcome (co-primary)** \| 1. Total duration of antibacterial prescription for the index visit \| Superiority analysis; includes number of days that antibacterials are prescribed during index hospitalization and at discharge (maximum 14 days); excludes antivirals, antifungals, anti-parasitics \| \| 2. Clinical outcomes by day 30 \| Non-inferiority analysis; composite binary endpoint (any of the following): a) Use of non-invasive ventilation (CPAP^c^ or BiPAP^d^) b) Use of mechanical ventilation or ECMO^e^ c) Readmission to hospital d) Death \| \| **Secondary outcomes** \| 1.Individual components of the composite clinical endpoint \| Each of the 4 adverse outcomes listed in primary outcome 2 \| \| 2. Seeking outpatient care and receiving antibacterial prescription by Day 30 \| At day 30 only \| \| 3.ICU^f^ admission and duration during index hospitalization \| Admission to ICU and duration in days \| \| 4.Use and duration of use of supplemental oxygen during index hospitalization \| Via nasal cannula, high flow nasal cannula, or face mask \| \| 5.Duration of non-invasive ventilation \| During index hospitalization \| \| 6.Duration of mechanical ventilation \| During index hospitalization \| \| 7.Antibacterial prescription \| On Day 1, 2, 3, at discharge, and by Day 30 \| \| 8.Total antibacterial exposure \| Total number of days antibacterials were prescribed during hospitalization and by Day 30 \| \| 9.Oseltamivir prescription \| On Day 1, Day 2, Day 3, at discharge, and by Day 30 \| \| 10.Total oseltamivir exposure \| Total number of days oseltamivir is prescribed during hospitalization and by Day 30 \| \| 11.SARS-CoV-2 antiviral prescription timing \| Day 1, Day 2, Day 3, at discharge, and by Day 30 \| \| 12.Physician adherence to eCDST^g^ diagnostic recommendations \| Within 24 hours and 48 hours (intervention group only) \| \| 13.Physician adherence to eCDST treatment recommendations \| Within 24 hours and 48 hours (intervention group only) \| \| **Exploratory Outcomes** \| 1.DOOR (Desirability of Outcome Ranking) level by Day 30 \| Ordinal scale (see Table 2) \| \| 2.Individual DOOR levels \| By Day 30 \| \| 3.Prescription of immunomodulatory therapy for SARS-CoV-2 \| Dexamethasone or tocilizumab on Day 1, Day 2, Day 3, and at discharge \|   ^c^continuous positive airway pressure, ^d^bilevel positive airway pressure, ^e^extracorporeal membrane oxygenation, ^f^intensive care unit  The clinical endpoint will be a binary endpoint that is a composite of adverse outcomes that could be attributed to withholding antibacterials. These outcomes would not be present at enrollment, and could occur anytime until Day 30. At least one of the following adverse outcomes should be present to meet this endpoint: 1) use of non-invasive ventilation (*i.e.*, continuous positive airway pressure [CPAP] or bilevel positive airway pressure [BiPAP]) for treatment of the acute illness), 2) use of mechanical ventilation (via endotracheal tube) or extracorporeal membrane oxygenation (ECMO), 3) readmission to hospital, 4) death  Day 1 will be day of enrollment, and participants will be followed through Day 30.  Secondary outcomes will include the following: individual components of the binary clinical outcomes in the co-primary endpoint ; receipt and duration of antibacterial and antiviral prescription; physician adherence to the RespiQuestAB diagnostic and treatment recommendations, in the intervention group,  The exploratory outcomes will be the following: the Desirability of Outcome Ranking (DOOR) level by Day 30 arranged in an ordinal scale as shown in Table 2; individual DOOR levels by Day 30; and prescription of immunomodulatory therapy for SARS-CoV-2 (*i.e.*, dexamethasone, tociluzumab) on Day 1, Day 2, Day 3, and at discharge. Qualifying symptoms for adequate clinical improvement per the DOOR are shown in Table 3, and solicited adverse events per the DOOR are shown in Table 4.  **Table 2**: Desirability of Outcome Ranking (DOOR) of clinical outcomes (from most to least desirable).   \|  \| Adequate clinical improvement (assessed at Day 30)* \| Solicited adverse events (assessed through Day 30)^+^ \| \| --- \| --- \| --- \| \| 1 \| Yes \| None \| \| 2 \| Yes \| Mild- severe (grades 1-3) \| \| 3  4 \| No  No \| None  Mild- severe (grades 1-3) \| \| 5 \| No, with outpatient visit for LRTI \| None or any grade \| \| 6 \| No, with subsequent hospitalization \| None or any grade \| \| 7 \| Death (any cause) \| -- \| \| ** Adequate clinical improvement includes ALL of the following:*  *- Improvement in at least one qualifying symptom present at enrollment (see Table 3) by at least one step.*  *- Absence of deterioration in any qualifying symptom (see Table 3), defined as worsening by at least one step of any qualifying symptom*  *- No medically attended visit for LRTI*  *^+^ Solicited adverse events include antibiotic-related side effects, as listed in Table 4* \| \| \|   **Table 3**: Qualifying symptoms for adequate clinical improvement (Table 2) and grading scale   \|  \| Mild \| Moderate \| Severe \| \| --- \| --- \| --- \| --- \| \| Cough \| Occasional coughing (less than hourly) \| Frequent coughing (1 or more times an hour and interfered with activity or sleep) \| Almost constant coughing (never free of cough or need to cough, made activity or sleep nearly impossible) \| \| Sputum production \| Noticeable as a problem but did not interfere with activity \| Caused a great deal of inconvenience \| An almost constant problem \| \| Chest pain \| Noticeable only when coughing \| Noticeable during deep breaths and when coughing \| Almost constant, present even when resting, without cough \| \| Difficulty breathing \| Noticeable during strenuous activity, such as going up a flight of stairs or walking more than a block on level ground \| Noticeable during light activity, or when washing or dressing \| Almost constant, present even when resting \|   **Table 4**: Solicited adverse events and grading scale   \|  \| Mild \| Moderate \| Severe \| \| --- \| --- \| --- \| --- \| \| Abdominal pain \| Mild or intermittent and did not interfere with daily activity \| Moderate or persistent and interfered with daily activity but did not necessitate a medical visit \| Prevented daily activity and resulted in medical visit \| \| Vomiting \| 1 episode/day \| 2-3 episodes/day but did not necessitate a medical visit \| Prevent daily activity and resulted in medical visit/ hospitalization \| \| Diarrhea \| Looser than normal stools occurring 3-6 times/day \| Looser than normal stools >6 times/day but did not necessitate a medical visit \| Bloody diarrhea or diarrhea that required clinical evaluation or hospitalization \| \| Allergic reaction \| New localized rash or itching without rash \| New diffuse rash covering multiple areas of the body but did not necessitate a medical visit \| New rash requiring clinical visit \| \| Candidiasis \| Mild mucocutaneous candidiasis, with no treatment \| Moderate mucocutaneous candidiasis and interfered with daily activity but did not necessitate a medical visit \| Severe mucocutaneous candidiasis; resulted in medical visit \| |
| Participant timeline | [#13](https://www.goodreports.org/reporting-checklists/spirit/info/#13) | Time schedule of enrolment, interventions (including any run-ins and washouts), assessments, and visits for participants. A schematic diagram is highly recommended (see Figure) | Page 17, Line 414-416 | Figure 4 presents a detailed participant timeline, developed in accordance with SPIRIT guidelines.  **Figure 4**: Schedule of enrolment, interventions, and assessments |
| Sample size | [#14](https://www.goodreports.org/reporting-checklists/spirit/info/#14) | Estimated number of participants needed to achieve study objectives and how it was determined, including clinical and statistical assumptions supporting any sample size calculations | Page 21-22, Line 471-488 | The trial is powered on both co-primary endpoints. For co-primary endpoint 1 (total duration of antibacterial prescription for the index visit), power calculations were performed using the R package swCRT design (39). We assume that 9 pairs of wards (clusters) will cross over to intervention in 3 steps (3 pairs of wards per step), leading to 4 total time periods (including baseline). We assume a type I error of 0.025 since this is a co-primary endpoint. We assume a standardized effect size ($\delta$) of 0.5. Based on prior data, we estimate the mean antibiotic days to be 3.6 (SD=3.9). Given this standard deviation, we estimate the unstandardized effect size ($\Delta$) would be $\frac{\Delta}{SD}=\delta\Rightarrow\Delta=\delta*SD\Rightarrow\Delta=3.9*0.5=$ 1.95 antibiotic days. Under these assumptions, we would have 81% power to detect this effect with 765 total subjects recruited, assuming 20% of subjects drop out of the study.  For co-primary endpoint 2 (composite of adverse outcomes that could be attributed to withholding antibacterials), power calculations were performed using the non-inferiority calculator in the artbin package in Stata. Since there are no non-inferiority power calculators for stepped wedge trials, we calculate non-inferiority under an individually randomized trial design and inflate the sample size for a design effect defined by an ICC of 0.05 and 9 clusters. We assume a type I error of 0.025 since this is a one-sided test. Under these assumptions, we have 90% power to conclude non-inferiority with a control period rate of 10% and a 10% non-inferiority margin with 765 total participants, assuming 20% dropout. |
| Recruitment | [#15](https://www.goodreports.org/reporting-checklists/spirit/info/#15) | Strategies for achieving adequate participant enrolment to reach target sample size | Page 12, Line 294-303 | Participants meeting the LRTI case definition will be approached by trained research staff within 48 hours of hospital admission to medical wards (as opposed to the Emergency Treatment Units or Outpatient Departments), since clinical decisions during hospitalization are made by ward physicians. The study will be explained in English or the local languages of Sinhala or Tamil by multilingual, trained research assistants.  Potential participants will be screened for eligibility by study personnel during hospital admission. If the patient meets all inclusion criteria and does not meet any exclusion criteria, they will be offered enrollment following informed consent. Written, informed consent in Sinhala, Tamil, or English will be obtained from all adult patients and the parents or guardians of patients 14-17 years of age. Assent will be obtained from patients 14-17 years of age. |
| **Methods: Assignment of interventions (for controlled trials)** | | | | |
| Allocation: sequence generation | [#16a](https://www.goodreports.org/reporting-checklists/spirit/info/#16a) | Method of generating the allocation sequence (eg, computer-generated random numbers), and list of any factors for stratification. To reduce predictability of a random sequence, details of any planned restriction (eg, blocking) should be provided in a separate document that is unavailable to those who enrol participants or assign interventions | Page 13-14, Line 326-338 | The sequence of allocation of the pairs of wards to the sequential implementation of the algorithm will be prepared by an unblinded statistician using a computer-generated random number table using R software (version 4.4.2). A constrained randomization scheme will be used to ensure that for each of the two smaller hospitals, the two clusters from that hospital do not end up in the same sequence (in order to maximize balance of possible hospital-level effects). This unblinded statistician will communicate the allocation sequence to the study team. Training for implementing the intervention will be conducted in the appropriate wards no sooner than two weeks before the intervention is implemented. Wards will be blinded to their randomization time point until training for the implementation of the RespiQuestAB begins on the ward, which will not be more than two weeks prior to the switch. During these two weeks, an orientation session regarding the study objectives, protocol, RespiQuestAB use, and ethical considerations will be provided to clinicians. Due to the nature of the intervention, the treating clinicians, study staff, and participants will not be blinded to allocation. |
| Allocation concealment mechanism | [#16b](https://www.goodreports.org/reporting-checklists/spirit/info/#16b) | Mechanism of implementing the allocation sequence (eg, central telephone; sequentially numbered, opaque, sealed envelopes), describing any steps to conceal the sequence until interventions are assigned | Page 14, Line 330-333 | This unblinded statistician will communicate the allocation sequence to the study team. Training for implementing the intervention will be conducted in the appropriate wards no sooner than two weeks before the intervention is implemented. |
| Allocation: implementation | [#16c](https://www.goodreports.org/reporting-checklists/spirit/info/#16c) | Who will generate the allocation sequence, who will enrol participants, and who will assign participants to interventions | Pages 13-14, Line 326-328 , Page 12, Line 297-298 | The sequence of allocation of the pairs of wards to the sequential implementation of the algorithm will be prepared by an unblinded statistician using a computer-generated random number table using R software (version 4.4.2).  Participants will be enrolled by trained research assistants. |
| Blinding (masking) | [#17a](https://www.goodreports.org/reporting-checklists/spirit/info/#17a) | Who will be blinded after assignment to interventions (eg, trial participants, care providers, outcome assessors, data analysts), and how | Page 14, Line 333-338 | Wards will be blinded to their randomization time point until training for the implementation of the RespiQuestAB begins on the ward, which will not be more than two weeks prior to the switch. During these two weeks, an orientation session regarding the study objectives, protocol, RespiQuestAB use, and ethical considerations will be provided to clinicians. Due to the nature of the intervention, the treating clinicians, study staff, and participants will not be blinded to allocation. The unblinded statisticians will be blinded after assignment to intervention. |
| Blinding (masking): emergency unblinding | [#17b](https://www.goodreports.org/reporting-checklists/spirit/info/#17b) | If blinded, circumstances under which unblinding is permissible, and procedure for revealing a participant’s allocated intervention during the trial | Page 14, Line 337-338 | N/A. Participants will not be blinded to allocation. |
| **Methods: Data collection, management, and analysis** | | | | |
| Data collection plan | [#18a](https://www.goodreports.org/reporting-checklists/spirit/info/#18a) | Plans for assessment and collection of outcome, baseline, and other trial data, including any related processes to promote data quality (eg, duplicate measurements, training of assessors) and a description of study instruments (eg, questionnaires, laboratory tests) along with their reliability and validity, if known. Reference to where data collection forms can be found, if not in the protocol | Page 14, Line 340-349 | The study procedures common to both groups at the initial visit will include administering a questionnaire and reviewing the medical record to obtain baseline information about socio-demographics, chronic medical conditions, medication use, vital signs, physical exam findings, and collection of samples, including 15 mL of blood, 1 nasal or nasopharyngeal sample, sputum sample (for future research), and a urine sample. All participants will be followed throughout their hospitalization, and information about test results, antimicrobials prescribed and received, and clinical outcomes will be recorded. All participants will participate in a follow-up visit (by telephone or in person) at day 30 ±3 days to obtain information about antimicrobial use, clinical outcomes, and post-discharge resource utilization. Home visits may be conducted at the participants’ homes to complete the follow up encounter, if needed. |
| Data collection plan: retention | [#18b](https://www.goodreports.org/reporting-checklists/spirit/info/#18b) | Plans to promote participant retention and complete follow-up, including list of any outcome data to be collected for participants who discontinue or deviate from intervention protocols | Pages 12-13, Line 304-307 | Retention will be enhanced by several methods. Participants will be provided with the option of following up by either telephone or in person, depending on preference and their available resources. There will be a 3-day grace period for the 30-day follow-up visit, during which participants can follow up. |
| Data management | [#19](https://www.goodreports.org/reporting-checklists/spirit/info/#19) | Plans for data entry, coding, security, and storage, including any related processes to promote data quality (eg, double data entry; range checks for data values). Reference to where details of data management procedures can be found, if not in the protocol | Pages 22-23, Line 495-504 | Study participant research data, which is for purposes of statistical analysis and scientific reporting, will be entered onsite into a secure Research Electronic Data Capture (REDCap) database and stored in a HIPAA-compliant online drive or secure cloud storage. This will not include the participant’s contact or other identifying information. Rather, individual participants and their research data will be identified by a unique study identification number. The study data entry and study management systems used by clinical sites and by Duke research staff will be secured and password protected. At the end of the study, all study databases will be de-identified and archived in the secure online cloud storage.  Samples and data will be stored using codes assigned by the investigators. Data will be kept in password protected computers. Only investigators will have access to the samples and data. |
| Statistics: outcomes | [#20a](https://www.goodreports.org/reporting-checklists/spirit/info/#20a) | Statistical methods for analysing primary and secondary outcomes. Reference to where other details of the statistical analysis plan can be found, if not in the protocol | Page 23-24, Line 508-543 | The primary analysis population will be the intention-to-treat population (based on whether a patient is enrolled from a cluster that is in usual care or intervention phase). A secondary per-protocol analysis will also be performed, based on whether patient receives usual care or intervention-based care.  The co-primary endpoint of total duration of antibacterial prescription for the index presentation will be treated as continuous and will be analyzed using a mixed effects model, to take into account dependence by time and by pairs of wards (clusters). Based on the expected impact of the RespiQuestAB on early antibacterial therapy and based on statistical considerations, we chose to use a maximum of 14 days for total duration of antibacterial therapy. A truncated window was chosen for logistical practicality, clinical significance, and to restrict the distribution of the outcome such that outliers do not overinflate the variance leading to reduced power. The statistical model will account for possible changes in time trend, include a fixed effect for the intervention, and include random intercepts for both the cluster (pairs of wards) and cluster-by-time interaction. The coefficient and confidence interval on the fixed effect for intervention will be used to assess effectiveness. The statistical model will also include random intercepts for both the cluster (pairs of wards) and cluster-by-time interaction. Both co-primary endpoints will be assessed at type I error of 0.025 to take into account multiple testing. Additionally, prior data indicates that we can expect about 18% of the patients to have zero antibiotic days. As a sensitivity analysis, we will also analyze this co-primary endpoint using a zero-inflated negative binomial mixed effects model with the same specification described above.  The co-primary endpoint of clinical outcomes (composite of adverse outcomes that could be attributed to withholding antibacterials) by day 30 will be treated as binary and will be analyzed using a regression model that similarly accounts for the dependent data structure. The statistical model will similarly account for possible changes in the underlying time trend and will include a fixed effect for the intervention, with the confidence interval on the fixed effect for intervention being used to assess non-inferiority. The intervention will be considered non-inferior to the control if the upper 97.5% confidence interval is less than the non-inferiority margin of 10%. Both co-primary endpoints will be assessed at type I error of 0.025 to take into account multiple testing.  All secondary and exploratory endpoints that are continuous will be analyzed using the same type of statistical model as is used to analyze the co-primary endpoint of total duration of antibacterial prescription. All secondary and exploratory endpoints that are binary will be analyzed using the same type of statistical model as is used to analyze the co-primary endpoint of clinical outcomes by day 30. Ordinal outcomes will be analyzed using an ordinal regression approach in the GEE framework. Secondary and exploratory endpoints will be assessed using only coefficients and confidence intervals. |
| Statistics: additional analyses | [#20b](https://www.goodreports.org/reporting-checklists/spirit/info/#20b) | Methods for any additional analyses (eg, subgroup and adjusted analyses) | Page 25, Line 553-558 | Subgroup analyses will be conducted to examine the differential effect of intervention on the co-primary endpoint of total duration of antibacterial prescription by sex, baseline disease severity, age group, duration of symptoms before randomization, and presence of comorbidities. Effect modification will be assessed by adding main effects and interactions between the aforementioned baseline covariates and intervention, with the p-value on the interaction testing for significant effect modification. A forest plot will be created to visually display the intervention effects within each level of the subgroup. |
| Statistics: analysis population and missing data | [#20c](https://www.goodreports.org/reporting-checklists/spirit/info/#20c) | Definition of analysis population relating to protocol non-adherence (eg, as randomised analysis), and any statistical methods to handle missing data (eg, multiple imputation) | Page 25, Line 544-552 | Although this study is a cluster-randomized stepped wedge trial (i.e., a different set of patients is followed up at each time point within each cluster), we anticipate some missing data on the primary endpoints since the patients will be followed for 30 days. The mixed effects model is valid under the missing at random framework. For both the mixed effects and GEE models, we will take into account missing data by first examining if there are any baseline (i.e., participant characteristic) variables associated with loss to follow-up. Then, we will adjust for these variables in the statistical models. As an additional sensitivity analysis, we will also adjust for any participant-level or cluster-level characteristics that are imbalanced by time that the clusters cross over. |
| **Methods: Monitoring** | | | | |
| Data monitoring: formal committee | [#21a](https://www.goodreports.org/reporting-checklists/spirit/info/#21a) | Composition of data monitoring committee (DMC); summary of its role and reporting structure; statement of whether it is independent from the sponsor and competing interests; and reference to where further details about its charter can be found, if not in the protocol. Alternatively, an explanation of why a DMC is not needed | Page 27, Line 595-602 | To oversee the TREAT-SL clinical trial, a Data Safety Monitoring Board (DSMB) will be established as an independent committee responsible for ensuring the safety of research subjects. It is anticipated that the DSMB will meet every 6 months to review the accumulating data. The DSMB will weigh any trade-offs between short-term versus long-term results. The DSMB would be asked to offer proper perspective on any therapeutic or diagnostic testing advances that may occur during the course of the trial. The DSMB will be comprised of a physician from Sri Lanka, a physician from the US, and a statistician. The DSMB will be independent from the sponsor and competing interests. A charter has been formed and appended as an annexure (additional file 1). |
| Data monitoring: interim analysis | [#21b](https://www.goodreports.org/reporting-checklists/spirit/info/#21b) | Description of any interim analyses and stopping guidelines, including who will have access to these interim results and make the final decision to terminate the trial |  | N/A. There will be no interim analyses, but the DSMB will review safety data every 6 months and make decisions to terminate the trial. |
| Harms | [#22](https://www.goodreports.org/reporting-checklists/spirit/info/#22) | Plans for collecting, assessing, reporting, and managing solicited and spontaneously reported adverse events and other unintended effects of trial interventions or trial conduct | Pages 26-27, Line 580-594 | We will capture the major potential adverse events related to antibacterial withholding in the safety component of the co-primary endpoint. The adverse outcomes that comprise the primary safety endpoint will be included in endpoint reporting, and thus will not be reported a second time as adverse events (AEs). However, those that are categorized as serious adverse events (SAEs) will be reported. Furthermore, these following additional events (not listed under the co-primary endpoint) will be captured and reported as AEs: the development of lung abscess/ empyema, and the development of pneumonia in non-pneumonia LRTI.  An AE will be defined as any untoward medical occurrence in a participant temporally associated with the study intervention or other aspects of study participation, but not necessarily considered related to the participant’s participation in the research study. An SAE will be defined as an untoward medical occurrence that: (1) results in death or (2) is life-threatening. Life-threatening will be defined as the following events: the development of septic shock (requiring vasopressor use), the need for mechanical ventilation, or the need for ECMO.  All adverse events (AEs and SAEs), as described above, occurring after study enrollment until the study day 30 follow-up visit will be recorded. |
| Auditing | [#23](https://www.goodreports.org/reporting-checklists/spirit/info/#23) | Frequency and procedures for auditing trial conduct, if any, and whether the process will be independent from investigators and the sponsor | Pages 27-28, Line 604-615 | Quality assurance personnel may conduct audits at the study sites. Audits will include, but not be limited to, examination of the audit trail of data handling and processes, standard operating procedures, presence of required documents, and the informed consent process. The investigators agree to accommodate and participate in audits conducted at a reasonable time and in a reasonable manner.  Regulatory authorities from Sri Lanka or elsewhere may also inspect the study site during or after the study. The study site should contact the study leadership immediately if this occurs and must fully cooperate with governmental audits conducted at a reasonable time and in a reasonable manner.  The study leadership and study site will provide access to all trial related sites, source data/documents, and reports for the purpose of monitoring and auditing by the sponsor, and inspection by local and regulatory authorities. |
| **Ethics and dissemination** | | | | |
| Research ethics approval | [#24](https://www.goodreports.org/reporting-checklists/spirit/info/#24) | Plans for seeking research ethics committee / institutional review board (REC / IRB) approval | Page 28, Line 617-623 | This study was approved by the Ethics Review Committee, Faculty of Medicine, University of Ruhuna, Sri Lanka (Reference no:2023/P/113) on 30^th^ November 2023 and the Duke University Institutional Review Board (IRB) (IRB no: Pro00114347) on 04^th^ September2024. Furthermore, this clinical trial was registered in clinicaltrials.gov (Clinicaltrials.gov identifier: NCT06331364) and in the Sri Lanka Clinical Trial Registry (Reference no: SLCTR/2024/019). Additionally, obtaining administrative clearance from the Education, Training, and Research (ET&R) unit of the Ministry of Health, Sri Lanka, was mandatory. |
| Protocol amendments | [#25](https://www.goodreports.org/reporting-checklists/spirit/info/#25) | Plans for communicating important protocol modifications (eg, changes to eligibility criteria, outcomes, analyses) to relevant parties (eg, investigators, REC / IRBs, trial participants, trial registries, journals, regulators) | Page 28, Line 623-625 | Any amendments to the protocol, other than administrative ones, will be approved by the University of Ruhuna ERC, Duke University IRB and SLCTR according to their procedures. |
| Consent or assent | [#26a](https://www.goodreports.org/reporting-checklists/spirit/info/#26a) | Who will obtain informed consent or assent from potential trial participants or authorised surrogates, and how (see Item 32) | Page 12, Line 297-298 ,  Page 12, Line 299-303 | The study will be explained in the local languages of Sinhala or Tamil or English by multilingual, trained research assistants.  Potential participants will be screened for eligibility by study personnel during hospital admission. If they meet all inclusion criteria and do not meet any exclusion criteria, they will be offered enrollment following informed consent. Written, informed consent in Sinhala, Tamil, or English will be obtained from all adult patients and the parents or guardians of patients 14-17 years of age. Assent will be obtained from patients 14- 17 years of age. |
| Consent or assent: ancillary studies | [#26b](https://www.goodreports.org/reporting-checklists/spirit/info/#26b) | Additional consent provisions for collection and use of participant data and biological specimens in ancillary studies, if applicable |  | N/A. However, During the conduct of the study, an individual participant can choose to withdraw consent to have biological specimens stored for future research. |
| Confidentiality | [#27](https://www.goodreports.org/reporting-checklists/spirit/info/#27) | How personal information about potential and enrolled participants will be collected, shared, and maintained in order to protect confidentiality before, during, and after the trial | Page 22, Line 490-504 | Participant confidentiality is strictly held in trust by the participating investigators, their staff, and the sponsor(s) and their agents. The study participant’s contact information will be securely stored at each clinical site for internal use during the study. At the end of the study, all records will continue to be kept in a secure location for at least 5 years beyond the conclusion of the study.  Study participant research data, which is for purposes of statistical analysis and scientific reporting, will be entered onsite into a secure Research Electronic Data Capture (REDCap) database and stored in a HIPAA-compliant online drive or secure cloud storage. These data will not include the participant’s contact or other identifying information. Rather, individual participants and their research data will be identified by a unique study identification number. The study data entry and study management systems used by clinical sites and by coordinating center will be secured and password protected. At the end of the study, all study databases will be de-identified and archived in the Duke Box folder.  Samples and data will be stored using codes assigned by the investigators. Data will be kept in password protected computers. Only investigators will have access to the samples and data |
| Declaration of interests | [#28](https://www.goodreports.org/reporting-checklists/spirit/info/#28) | Financial and other competing interests for principal investigators for the overall trial and each study site | Page 32, Line 675-676 | The authors have no personal, financial, or institutional interest in any of the materials, or devices, being used in this trial. |
| Data access | [#29](https://www.goodreports.org/reporting-checklists/spirit/info/#29) | Statement of who will have access to the final trial dataset, and disclosure of contractual agreements that limit such access for investigators | Pages 22-23, Line 495-502 | Data collected for this study will be analyzed and stored in a secure online drive, in collaboration with co-investigators from the University of Ruhuna. After the study is completed, the de identified, archived data will be transmitted to and stored in a secure cloud storage, under the supervision of PI. |
| Ancillary and post trial care | [#30](https://www.goodreports.org/reporting-checklists/spirit/info/#30) | Provisions, if any, for ancillary and post-trial care, and for compensation to those who suffer harm from trial participation | Page 14-15, Lines 349-351 | N/A. As patients will only remain in study until the last follow-up at day 30. Free care is available at the three public hospitals, as it is to all patients. |
| Dissemination policy: trial results | [#31a](https://www.goodreports.org/reporting-checklists/spirit/info/#31a) | Plans for investigators and sponsor to communicate trial results to participants, healthcare professionals, the public, and other relevant groups (eg, via publication, reporting in results databases, or other data sharing arrangements), including any publication restrictions | Page 29-30, Line 648-652 | The outcomes of this clinical trial will be published in peer-reviewed medical or public health journals with open access to ensure accessibility to a wide range of the research community, especially in LMICs. Manuscripts will also be submitted for digital archiving in PubMed Central upon acceptance for publication. Additionally, results will be presented at local and international conferences, and will also be shared with the clinicians and hospitals involved in the trial. |
| Dissemination policy: authorship | [#31b](https://www.goodreports.org/reporting-checklists/spirit/info/#31b) | Authorship eligibility guidelines and any intended use of professional writers | Page 33, Line 685-686 | There will be no use of professional writers.  All study team members who contributed significantly to the development or implementation of the study, or analysis or publication of the study results, will be eligible for authorship. |
| Dissemination policy: reproducible research | [#31c](https://www.goodreports.org/reporting-checklists/spirit/info/#31c) | Plans, if any, for granting public access to the full protocol, participant-level dataset, and statistical code | Page 30, Line 653-656 | The full protocol will be provided as a supplementary document upon publication of the results. We have not received permission from the regulatory authorities to share participant-level datasets. However, we may be able to publish the statistical code as supplementary documentation upon publication of the results. |
| **Appendices** | | | | |
| Informed consent materials | [#32](https://www.goodreports.org/reporting-checklists/spirit/info/#32) | Model consent form and other related documentation given to participants and authorised surrogates | Page 32, Line 671-673 | Informed consent forms were approved by the Ethical Review Committee, Faculty of Medicine, University of Ruhuna, IRB-Duke University, SLCTR and ET&R, and will be used in the trial. Informed consent forms can be provided by the principal investigator upon request. |
| Biological specimens | [#33](https://www.goodreports.org/reporting-checklists/spirit/info/#33) | Plans for collection, laboratory evaluation, and storage of biological specimens for genetic or molecular analysis in the current trial and for future use in ancillary studies, if applicable | Page 23,Line 504-506 | With the participant’s approval and as approved by local IRBs, blood, sputum, urine, and nasal/ nasopharyngeal samples will be stored at the University of Ruhuna . These samples could be used for research into the etiology of infection and response to infection. |

It is strongly recommended that this checklist be read in conjunction with the SPIRIT 2013 Explanation & Elaboration for important clarification on the items. Amendments to the protocol should be tracked and dated. The SPIRIT checklist is copyrighted by the SPIRIT Group under the Creative Commons “[Attribution-NonCommercial-NoDerivs 3.0 Unported](http://www.creativecommons.org/licenses/by-nc-nd/3.0/)” license. This checklist can be completed online using https://www.goodreports.org/, a tool made by the EQUATOR Network in collaboration with Penelope.ai
